# Unclonable human-invisible machine vision markers leveraging the omnidirectional chiral Bragg diffraction of cholesteric spherical reflectors

**DOI:** 10.1038/s41377-022-01002-4

**Published:** 2022-10-25

**Authors:** Hakam Agha, Yong Geng, Xu Ma, Deniz Işınsu Avşar, Rijeesh Kizhakidathazhath, Yan-Song Zhang, Ali Tourani, Hriday Bavle, Jose-Luis Sanchez-Lopez, Holger Voos, Mathew Schwartz, Jan P. F. Lagerwall

**Affiliations:** 1 University of Luxembourg, Department of Physics & Materials Science, 1511 Luxembourg, Luxembourg; 2 University of Luxembourg, Interdisciplinary Centre for Security, Reliability and Trust (SnT), 1855 Luxembourg, Luxembourg; 3 University of Luxembourg,University of Luxembourg, Department of Engineering, L-1359 Luxembourg, Luxembourg; 4 New Jersey Institute of Technology, College of Architecture and Design, University Heights, Newark, NJ USA

**Keywords:** Optical data storage, Liquid crystals, Imaging and sensing

## Abstract

The seemingly simple step of molding a cholesteric liquid crystal into spherical shape, yielding a *Cholesteric Spherical Reflector* (CSR), has profound optical consequences that open a range of opportunities for potentially transformative technologies. The chiral Bragg diffraction resulting from the helical self-assembly of cholesterics becomes omnidirectional in CSRs. This turns them into selective retroreflectors that are exceptionally easy to distinguish—regardless of background—by simple and low-cost machine vision, while at the same time they can be made largely imperceptible to human vision. This allows them to be distributed in human-populated environments, laid out in the form of QR-code-like markers that help robots and Augmented Reality (AR) devices to operate reliably, and to identify items in their surroundings. At the scale of individual CSRs, unpredictable features within each marker turn them into Physical Unclonable Functions (PUFs), of great value for secure authentication. Via the machines reading them, CSR markers can thus act as trustworthy yet unobtrusive links between the physical world (buildings, vehicles, packaging,…) and its digital twin computer representation. This opens opportunities to address pressing challenges in logistics and supply chain management, recycling and the circular economy, sustainable construction of the built environment, and many other fields of individual, societal and commercial importance.

## Abstract (web summary)

Cholesteric Spherical Reflectors (CSRs) are omnidirectional retroreflectors that are selective in circular polarization and wavelength, enabling human-invisible fiducial markers for applications in robotics, augmented reality, anti-counterfeiting, circular economy and more

## Graphical abstract

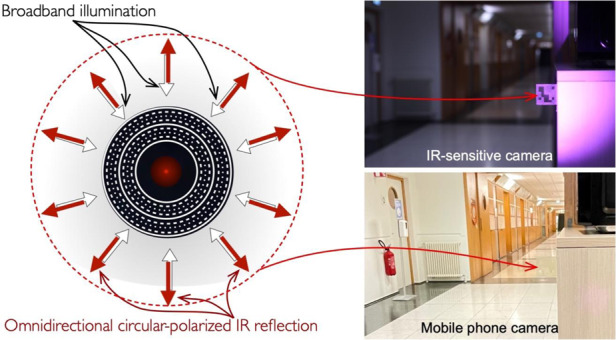

## Introduction

Since the information technology revolution, an increasingly rich digital world exists in parallel to, and separated from, our physical reality, but these two worlds are now on a clear path to merging^1–4^. Concepts like Industry 4.0, the Metaverse and the Internet of Things (IoT) all embrace a single mixed reality that seamlessly blends physical and digital aspects^5–10^. The digital world is coming to us in the form of 3D-printing, wearable devices, Augmented Reality (AR), self-driving vehicles and other robots deployed where humans live, work and play^1,11–16^. Meanwhile, previously purely physical items enter the digital world via IoT devices like wireless transceivers or RFID chips^5,17,18^. Even humans are now becoming’ digitally twinned’^19–21^, a development we all experienced when the COVID-19 pandemic made us subject to digital gate keepers requesting our personal QR-codes at airports, restaurants, events etc. Central to this development on the digital side is blockchain^5,22^ which enables the unique identification and reliable tracking of digital assets. Giving physical objects their unique digital twin representation, as in Building Information Modeling (BIM), is an enormously powerful concept applicable across vastly different fields, from thwarting counterfeiting^2,23–25^ to fighting climate change by monitoring the true environmental footprints of products and buildings^26,27^. Unfortunately, a suitable link between the physical and digital worlds, that is unobtrusive, robust and unforgeable while applicable to any relevant physical item—active or passive—is still largely missing^28^. This prevents the full potential of digital twinning from being unleashed, since users cannot be certain that the digital information accurately corresponds to the physical entity they are dealing with.

We believe that the material in focus of this article, *Cholesteric Spherical Reflectors*, or CSRs for short, can play a significant role in providing the missing link^28^. With CSRs we can encode information onto surfaces in the form of QR-code-like markers that are very easily readable by robots and AR devices using simple and low-cost optical components, allowing them to reliably identify each object carrying such a marker as well as its position and orientation. Since the markers can be made very difficult to detect by the human eye, they can be ubiquitously deployed even in human-populated spaces with minimum impact on the environment as we experience it^29^. As robots and AR devices are simultaneously present in the physical and digital worlds, they can update the digital twin if they detect changes in the physical world marked up with CSR patterns. Robots can also manipulate physical objects identified through their CSR markers to carry out changes that were planned in the digital twin. Moreover, the rich optical characteristics of CSRs^30–40^ gives each marker a unique fingerprint that allows the reading unit to distinguish an original from a fake^28,35,38,41,42^, or to track a certain object throughout its lifetime. This is of great use for increased supply chain transparency and circular economy solutions involving recycled materials and components.

To make these arguments we start out from two prior articles where early versions of these concepts were first presented^28,38^, adding recent advances that demonstrate the plausibility and scalability of the proposed technologies, and placing the discussion in the context of important recent developments in each application domain considered. After summarizing the defining characteristics of cholesteric liquid crystals and introducing CSRs in their two main versions (beads and shells), we show how the spherical modulation of the self-organized cholesteric order gives rise to unique optical properties that can become extraordinarily powerful. We demonstrate, for the first time, how machine vision can be designed to detect and read CSR markers in real time with great clarity, even when they are designed for minimum detection by the human eye. This allows us to discuss the endless range of intriguing application opportunities that CSR markers open. Specifically, we argue that CSR markers offer a cutting-edge solution to assist the localization and analysis of the surroundings of robots and AR devices^43^, with particular benefits in construction of the built environment^44^; to support circular economy solutions, simplify recycling and enable reliable track-and-trace functionality across complex supply chains^27,45^; and to support the increasingly urgent fight against counterfeit and other substandard products and materials^35,42,46^, which pose a global threat not just to the economy but even more so to health and the environment^23,24^. The article is complemented by rich Supplementary Information that provides more detail on selected issues.

### What are cholecteric spherical reflectors (CSRs)?

#### Cholesteric liquid crystals and their peculiar reflection optics

**Fig. 1:**
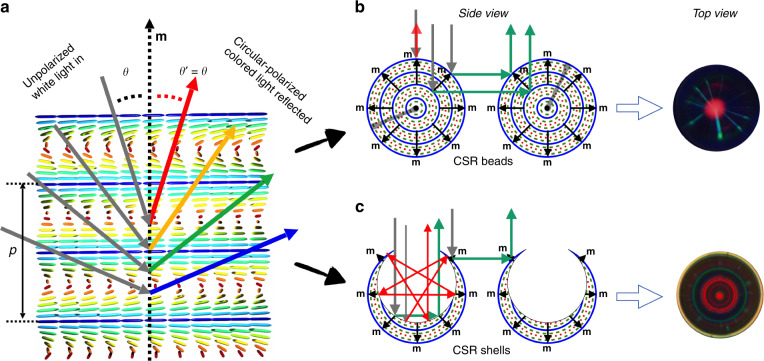
Chiral Bragg diffraction from flat and spherical cholesterics. **a** Schematic drawing of the helically modulated structure (rods represent **n**, color-coded blue→red for **n** parallel→perpendicular to the image plane) of a cholesteric liquid crystal and the resulting wavelength- and polarization-selective reflection due to Bragg diffraction. Grey arrows represent unpolarized white incident light at different incidence angles θ with respect to **m**, each corresponding reflection colored to the wavelength that is Bragg-diffracted at that θ. Since the helix is drawn right-handed, the reflected light would have right-handed circular polarization. **b** Schematic cross section of CSR beads (or droplets) with radial helix orientation, a point defect (black) at the core, and arbitrarily oriented Frank-Pryce defect lines (semi-transparent grey). If the retroreflection wavelength is red (central spot in top view), the cross communication is green. Since the latter happens throughout the bead at different depths, radial lines result^33^. **c** Schematic cross section of polymerized and punctured CSR shells, defect-free since shells have no point defect and the Frank-Pryce defect line is removed upon puncturing. Shells illuminated from the thin side primarily produce internal selective reflection rings^39^, but some cross communication (spots rather than lines, due to the isotropic core^35^) may also be seen, as in the top view example (unpunctured shell). Color coding of director orientation in (**b**-**c**) is as in (**a**)

The hallmark of cholesteric liquid crystals^47–49^ is their ability to self-organize their (most often) rod-shaped molecules with two types of long-range orientational order that are mutually orthogonal. First, the molecules align along a common direction (the director, **n**), as in non-chiral *nematic* liquid crystals, frequently used in display devices. This gives rise to anisotropic properties, in terms of optics making the phase birefringent with **n** the optic axis in a non-chiral nematic. Normally the birefringence is positive, ∆*n* = *n*_||_ −*n*_⊥_ *>* 0, with a greater refractive index *n*_||_ for light polarized along **n** than the index *n*_⊥_ experienced for perpendicular polarization. The second type of long-range order is unique to cholesterics, and this amounts to a helical modulation of the director along an axis **m** perpendicular to **n**, see Fig. 1a. The helix has a well defined pitch (or period) *p* and can be either right- or left-handed.

Because its self-assembled structure is equivalent to chirally modulated nematic order, a cholesteric liquid crystal can also be referred to as *chiral nematic*. The term cholesteric (referring to the first discovery in a cholesteryl derivative^50^) is often preferred when *p* is short enough to significantly alter the physical properties of the phase^49^. The most important such alteration occurs in the optical properties when *p* approaches the wavelength of light, *λ*^*LC*^, as measured within the liquid crystal^48^. If *p* > *λ*^*LC*^ the director remains the optic axis, which is thus modulated with the helix. This gives rise to characteristic fingerprint textures when observing such phases perpendicular to **m** in a microscope, and it leads to scattering of visible light. In contrast, when *p* ≤ *λ*^*LC*^, light cannot resolve the helix and it is instead the helix axis **m** that takes the role of optic axis, the birefringence becoming negative (assuming that the phase is made up of molecules similar to those in Supplementary Note 1, producing ∆*n*^*nh*^ > 0 in the absence of helix; the superscript ’nh’ stands for non-helical).

The most striking effect of such ’short-pitch’ cholesterics is a strongly colored and circularly polarized iridescent reflection. The reason is that the eigenmodes for visible light in the liquid crystal are now circularly polarized, one with the same handedness as the helix, the other with the opposite handedness, and the former cannot propagate through the cholesteric if *λ*^*LC*^ ≈ *p*^51^; instead it is ’selectively reflected’. Quantitatively, the selective reflection is described as Bragg diffraction of light that is circularly polarized with the same handedness as the helix, with wavelength in a band centered around:

1 $$\lambda ^{LC} = p\cos \theta$$

where θ is the angle of incidence of light, within the liquid crystal, with respect to the helix axis **m** (see Fig. 1a). Note that θ will be different from the angle of incidence *α* at which light enters a medium containing CSRs from air, due to refraction at the interface. As will become clear below, the binder surrounding CSRs must be index matched to the CSRs, hence refraction at the internal interface is negligible, but at the air interface it has impact. Distinguishing between *α* and θ is thus important, and awareness of the difference can be helpful in maximizing the performance of CSRs in applied contexts, as demonstrated by Geng et al.^29^.

Equation (1) is Bragg’s law for first-order diffraction; higher orders are allowed at non-zero θ, but they are very weak. The equation shows that the maximum Bragg diffraction wavelength is *λ*_0_^*LC*^ = *p*, occurring for θ = 0. We call it the *retroreflection* wavelength, since this corresponds to a light propagation direction (within the cholesteric) that is parallel (or antiparallel) to the helix, which reflects the selected component back to the source. This holds even when considering the impact of refraction at the interface between air and a medium in which CSRs are embedded^29^. The predicted width of the selective reflection band is ∆*λ*^*LC*^ = *p*∆*n*^*nh*^. Ideally, all circularly polarized light with the handedness of the helix is reflected across the full bandwidth^52^, but in practice, many samples show a gradual decay of reflectance for wavelengths departing from *λ*_0_^53^. The selectivity of reflecting only one circular polarization, as determined by the unichiral make-up of the phase, makes cholesterics stand out from other structurally colored materials, such as periodic multilayer stacks of materials with different refractive indices^54^, block copolymers^55,56^ or colloidal crystals^57^. As we will see below, this property is key to the application opportunities that we will discuss in this paper.

#### Cholesteric spheres as omnidirectional chiral Bragg reflectors

CSRs are obtained by producing spherical droplets of cholesteric liquid crystal with tangential boundary condition for **n**, thereby ensuring a radial orientation of **m** (the method is described in Supplementary Note 2). Since **m** is the symmetry axis by which the effect of Bragg diffraction is determined, a well-ordered CSR is a spherically symmetric, or omnidirectional, Bragg reflector^30^. CSRs can be made, first, as spherical droplets consisting solely of cholesteric liquid crystal, often polymerized into solid beads after production. The ideal structure is that depicted in Fig. 1b. Second, we can make CSR shells by including a droplet of isotropic liquid (typically a water solution) *inside* each cholesteric droplet, **n** being parallel to the interface both at the outer and the inner boundary, see Fig. 1c. Because of density mismatch, the inner droplet rises to the top or sinks to the bottom by gravity, breaking the spherical symmetry. CSR shells thus typically have a well-defined *cylindrical* symmetry axis, extending from the thinnest to the thickest point. The drawing in Fig. 1c ignores the subtle bend in **m** that is expected from the non-concentric inner and outer boundaries.

**Fig. 2:**
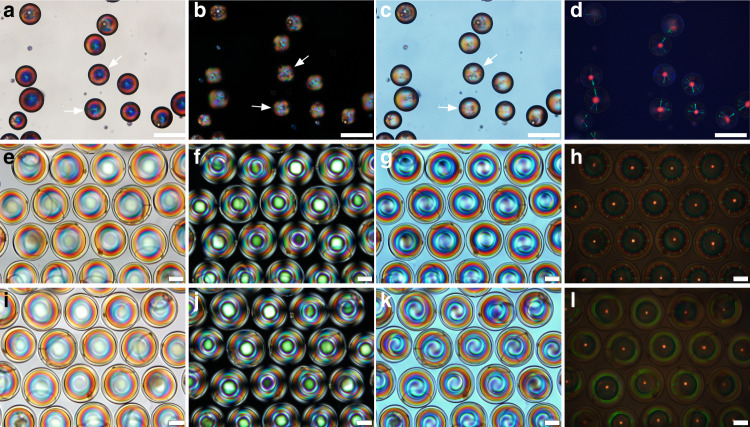
Comparison of CSR beads and shells. CSRs with red retroreflection are imaged in transmission without analyzer (**a**/**e**/**i**) and between crossed polarizers (**b**/**f**/**j**), with a *λ/*4 plate inserted in (**c**/**g**/**k**), and in reflection between crossed polarizers (**d**/**h**/**l**). The upper row shows polymerized beads whereas the lower two rows show polymerized shells (in both cases, the CSRs were annealed several days prior to polymerization), imaged from the thick (**e**–**h**) and from the thin (open) side (**i**–**l**), respectively. The beads are immersed in water whereas the shells are in cured NOA160 glue to allow imaging from both directions; density mismatch orients the shells with their opening upwards in liquid NOA160. When imaged from the thick side, cross communication between the CSRs is seen along the shell peripheries, whereas imaging from the open side shows the internal ring reflection. The central red retroreflection spot is clearly seen in all cases, external in (**d**/**h**) and internal in (**l**). While the reflection pattern looks good for beads as well as shells, the transmission images clearly reveal that the beads are less well aligned, they scatter light significantly more, and they also exhibit the characteristic Frank-Pryce line defect emanating from the center (highlighted with white arrows for two beads), further adding to the scattering. Scale bars represent 100 µm

To make CSRs that can be practically useful, one generally polymerizes and crosslinks the liquid crystal into a solid^58–65^, although it is also possible to keep it fluid by containing it inside a rubbery or solid polymeric shell^66–70^. A problem with droplets/beads is that topological defects cannot be avoided: because all possible orientations of **m** overlap at the core of a sphere with radially aligned helix, each CSR droplet has a point defect at its center^71–76^, depicted as a black circle in Fig. 1b. Moreover, topology also requires that there is a total defect sum of +2 in every spherical surface with **n** in the plane of the surface^36,77^. Since a cholesteric droplet/bead with radial **m** consists of an infinite set of concentric such surfaces, it must have a + 2 defect line extending from the center to the outer boundary in one direction, a configuration often referred to as the Frank-Pryce +2 defect line^72,74,75,77,78^. In Fig. 1b we have drawn it as a semi-transparent grey line. Both the central core defect and the Frank-Pryce +2 defect line can easily be detected in polarizing optical microscopy (POM), see Fig. 2a–c which shows polymerized CSR beads with ∼60 *µ*m diameter in transmission without analyzer (a) and between crossed polarizers, without (b) and with (c) a *λ/*4 plate inserted. Each bead shows a characteristic radial color profile and strong scattering around the core defect in (a). The visibility of the Frank-Pryce defect line is different from one bead to the next, since its orientation is arbitrary. We have highlighted it for two beads where the line is largely in the imaging plane, making it particularly visible.

An additional problem with CSR droplets/beads is that the absence of an internal interface that aligns **n** often leads to poorer control of **m**. While annealing may improve the situation, it can take many days and the alignment often gets even worse upon polymerization^58^. The annealing time is reduced and the overall alignment quality improved by using very small cholesteric droplets, about 5–10 *µ*m in diameter^59,70^. This comes at the cost, however, of an increased relative impact of the defects, which retain their scale regardless of droplet diameter. The overall effect of defects and imperfect internal order is indiscriminate light scattering, making it more challenging to hide CSR beads from detection by human eye sight. It may thus seriously limit their applicability in the technologies discussed towards the end of the paper.

Cholesteric shells have no central point defect since the core is replaced by the inner isotropic liquid droplet. They still have the Frank-Pryce defect line, but the liquid crystal localizes it to the thinnest side of the shell to minimize free energy^79^. When a CSR shell is polymerized, the resulting shrinkage of the cholesteric phase causes frustration since it surrounds an incompressible isotropic core (Supplementary Note 2). The consequence is that the shell bulges out at the thinnest point, where it breaks during a subsequent acetone rinse^29^. This yields CSR shells with a single hole at the thinnest side, as sketched in Fig. 1c. An idealized polymerized CSR shell is thus defect-free: it never contained a core defect and the Frank-Pryce +2 defect line is removed when the shell is punctured.

Shells are also much easier and faster to anneal than droplets of comparable size, for two reasons. First, the inner interface to the internal isotropic droplet provides an important additional aligning agent, reinforcing the radial helix orientation. The dual-sided confinement also minimizes the orientation loss upon polymerization^35^. Second, since the liquid crystal is not impermeable to water, an osmotic pressure set up by a solute in the internal droplet that is not present in a surrounding water phase will drive water into the shell, making it thinner and larger^80^. When applied to cholesteric shells, this can reduce the annealing time significantly.

The optical quality of shells is thus better than of beads, as demonstrated in Fig. 2. While the birefringence still makes shells easy to detect using high-quality optical microscopy (e/i), the overall scattering is much less than for beads. It is challenging, however, to make shells that are small, current production technologies typically yielding diameters in the 100–300 *µ*m range. This complicates automated deposition of CSR shells into patterns, as standard printing technologies handle particles larger than a few microns poorly. Small CSR beads and larger CSR shells may thus have their respective ideal application contexts.

The most interesting aspect of CSRs is, of course, their selective reflection behavior. In a POM investigation, the retroreflection is seen as a circular spot at the center of each CSR, see Fig. 2d/h/l. This is where the light from the microscope lamp hits the CSR normal to its boundary and along **m**. For shells viewed with their opening upwards (Fig. 2l) this spot originates at the shell inside, at its thickest point, reflecting the light back in the same way as do beads, or as do shells externally when viewed with their openings down (Fig. 2d/h). In contrast to the appearance in transmission, there is little impact of defects and imperfect helix orientation in CSR beads in the crossed polarizer reflection image (Fig. 2d). This is because it is enough with about 5–10 helix pitches of well-aligned cholesteric to produce the complete selective reflection^81^, and the first few microns from the outer surface often have good radial helix orientation. Around the central spot we notice multicolored patterns in all CSRs, looking quite different for beads and shells, in the latter case also changing depending on observation direction. We will now explain these intriguing patterns.

### CSRs as photonic cross communication network nodes and as photonic echo chambers

The striking pattern of green lines and spots in Fig. 2d/h is a result of *photonic cross communication* between adjacent CSRs^32^, illustrated in the side view schematics in Fig. 1b. As shown in Fig. 1a, light that does not propagate along **m** is not retroreflected but reflected away from the helix by the same angle θ^′^ as the incidence angle θ. When light is shone vertically on a sphere, as in a typical reflection POM investigation of CSRs, most light reflected in this way never comes back to the objective and is thus undetected by the camera. However, when we have multiple CSRs of similar size in the plane, light hitting one CSR at θ = 45^°^ is reflected horizontally into the sample plane until it hits another CSR, also at θ = 45^°^. This CSR will thus reflect the light back up to the objective, as illustrated in Fig. 1b. Each CSR becomes a node in an intricate photonic cross communication network with channels that are blue-shifted compared to *λ*_0_ as quantified by Eq. (1). With θ = 45^°^ and red *λ*_0_ we get the green lines and spots in Fig. 2d/h. Shells show spots since light entering a shell at an angle different from 45^°^ to the boundary normal will reach the isotropic interior before encountering a region with **m** at the required θ = 45^°^ to the propagation direction. The situation is different for droplets and beads^33^: as illustrated with the central grey vertical arrow of incoming light in Fig. 1b, such a light beam continues into the bead until it eventually is Bragg diffracted horizontally by an *interior* segment where θ = 45^°^. This is why CSR beads and droplets show radial cross communication *lines*, extending from the core to the perimeter with slightly different focus, as shown in the example photo on the right in Fig. 1b.

The network is actually more complex than suggested by the drawing in Fig. 1b, depicting only the dominant direct CSR–CSR cross communication mode. Further modes are enabled by total internal reflection events at the binder–air interface^32^, by the spread in beam direction enabling communication between CSRs with different *p*^35^, and by communication paths involving more than two CSRs^37^. Since all cross communication is blue-shifted from *λ*_0_, it can significantly influence the apparent color, causing a mixing of long and short wavelengths and revealing CSRs designed for invisible retroreflection in the near-IR^29^.

For CSR shells observed along the symmetry axis from the thin side, an additional mode of retroreflection exists, blue-shifted compared to *λ*_0_^39^. As mentioned above and illustrated in Fig. 1c, light illuminating a shell from the thin side will enter *into* the isotropic interior before it experiences selective reflection, but this eventually happens when it reaches the thick side from the inside. In addition to the central retroflection, there are certain off-center distances that initiate a series of multiple internal reflections that eventually end with light being sent back up vertically until it reaches the objective and is thus picked up by the camera. The Bragg diffraction angle θ increases with distance from the center, hence the blue shift is stronger the further from the center that the light hits the bottom inside. Two such internal selective reflection paths of this photonic echo chamber phenomenon are illustrated in Fig. 1c. Because of the cylindrical symmetry of shells and the observation along the symmetry axis, the different paths fulfilling the criterion for retroreflection by multiple internal reflections produce a sequence of concentric colored circles, increasingly blue-shifted with increasing radius. The opening in polymerized shells removes the inner circles, hence the example micrograph shown on the right in Fig. 1c is obtained with a shell prior to polymerization. Some photonic cross communication between the CSR shell outsides may take place also in this configuration, a few spots seen together with the circles in the example photo (digitally enhanced to make both rings and spots clearly visible). Often the ring-shaped internal reflections dominate the behavior when shells are viewed from the thin side, while photonic cross communication dominates when they are viewed from the thick side, as demonstrated in the lower two rows of Fig. 2.

### Macroscopic optical behavior of CSRs in comparison to flat cholesteric films

So far, most studies of the optics of CSRs utilized polarizing optical microscopy, typically with illumination along the viewing direction, thus basically probing retroreflection. While these investigations have been instrumental in developing a deeper understanding of the near-field optical response of CSRs, the ambition to apply CSRs for encoding information for robotics and AR requires systematic investigations also on macroscopic scale, under varying illumination conditions. To fully appreciate the unique far-field behavior of CSRs, it is useful in this context to compare directly with the behavior of flat cholesteric films. Therefore, to further demonstrate the optics illustrated in Fig. 1a in practice, we prepare three flat samples as reference of polymerizable cholesteric between a black painted microscope slide and cover slips, with red, green and blue retroreflection color, respectively. The helix is right-handed for red and green and left-handed for blue retroreflection, and **m** is predominantly perpendicular to the film plane. Once a macroscopically uniform optical behavior is confirmed we cure each sample into a solid film by UV irradiation. These samples are compared to three wells of similar size on black background that are filled with suspensions of polymerized CSRs (some beads but mainly shells) in UV-curable glue (Norland Optical Adhesive, NOA, 160) that is close to index-matched to the CSRs. This binder has the advantage that it can be manipulated as a liquid as long as it is not exposed to light with wavelengths shorter than those of yellow light, allowing us to deposit a suspension of CSRs dispersed in NOA160 as we wish. We then turn the binder into a solid film by UV-curing, making the particular CSR arrangement permanent. We tune *p* of the CSRs to achieve near-IR ($\lambda _0^{air} \approx 0.75$µm), green and blue retroreflection, respectively. The former are right-handed while the latter are left-handed.

**Fig. 3:**
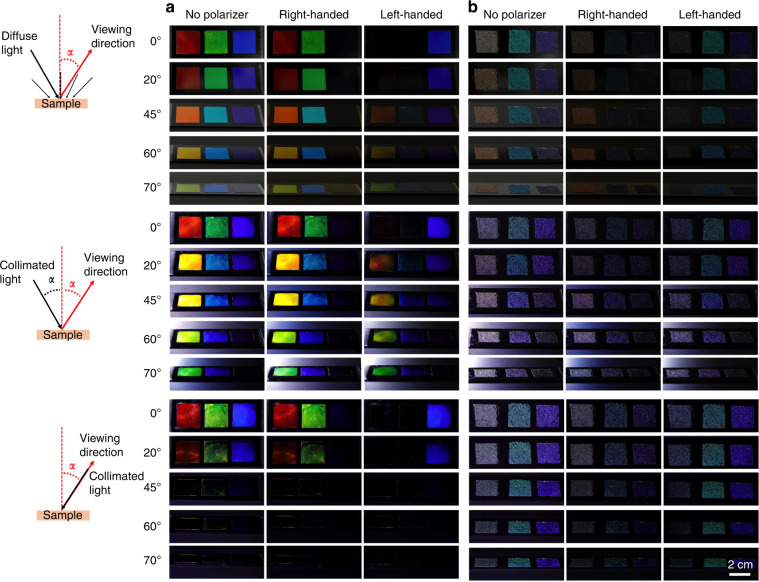
Comparison of reflection behavior of flat cholesteric films and densely packed CSRs on black background. Three flat films of polymerized cholesteric liquid crystal (**a**) with vertical helix, with red, green and blue retroreflection color, respectively, are compared to three samples with close-packed CSRs (**b**) in cured NOA160 glue, with near-IR, green and blue retroreflection, respectively. The red and green films and the near-IR CSRs have right-handed helix while the blue film and the green and blue CSRs have left-handed helix. Three white-light illumination and full-color imaging configurations are compared, as illustrated in the schematics on the left; top: diffuse outdoor illumination around noon on a cloudy day; middle: collimated light along a direction mirroring the imaging direction, the mirror plane containing the sample normal; bottom: collimated light parallel to the imaging direction. For each configuration, five imaging angles *α* are compared, without polarizer and through right- and left-handed circular polarizer, respectively

All samples are photographed at normal incidence and at four different angles *α* of inclined incidence, without polarizer and through right- and left-handed polarizer, respectively, see Fig. 3. Note that, because the refractive index of air is *n* = 1, much less than the *n* ≈ 1.6 of the samples, *α* ≥ θ, since the former angle refers to the normal of the sample–air interface while the latter angle refers to the cholesteric helix axis. We do the experiments with three different illumination conditions: (1) diffuse white light (outdoor photography on a cloudy day around noon), (2) illumination by collimated white light along the opposite direction of the imaging direction, with the same angle of incidence, and (3) illumination by collimated white light along the imaging direction. The reflection response of the polymerized flat cholesteric films is shown in the first set of photos (Fig. 3a) whereas that of the CSR-filled wells under identical imaging conditions is shown in the second set (Fig. 3b). We start by discussing the flat films.

As the viewing angle *α* is increased from normal to the sample, the apparent color of all samples blue shifts, as expected from Eq. (1) since increasing *α* also means increasing θ, with the effect that the blue sample appears almost uncolored for *α* > 60^°^, since the reflection wavelength has then shifted to the ultraviolet part of the spectrum. The samples with red and green *λ*_0_ remain colored until *α* ≈ 70^°^, the former ending up greenish and the latter blue. However, the color is not as pure as Eq. (1) would suggest, because we have diffuse illumination, with light incident along all directions against the samples, in which there is some variability in the orientation of **m** (see microscopic characterization in Supplementary Fig. 3a, g, m). We thus get a mixture of nearby reflection colors rather than pure spectral colors, explaining the brownish character of the sample that was red at *α* = 0^°^.

This is in stark contrast to the next illumination condition, when collimated light is shone onto the sample along the angle −*α*. The reflection colors are now close to the spectral colors expected from Eq. (1), because even if there is some variation in **m**, the narrow cone of light incidence selects only the parts of the sample with vertical **m** to yield Bragg diffraction that is imaged by the camera. While the left-most sample (red *λ*_0_) was orange at *α* = 45^°^ and brown at *α* = 60^°^ under diffuse illumination, it is now bright yellow and approaching green, respectively, at these viewing angles. Also the middle sample (green *λ*_0_) appears different, with a clear blue color at *α* ≈ 20^°^ and *α* ≈ 45^°^ rather than green and cyan, as was the case under diffuse light. With the right-most sample (blue *λ*_0_) the difference is less striking since *p* is here so short that the only visible selective reflection is in the blue–violet range.

The final illumination condition, with light along the viewing direction, leaves all flat film samples dark at *α* > 20^°^. This demonstrates that flat cholesterics are not omnidirectional retroreflectors, as indeed expected. They only exhibit retroreflection at normal incidence. For other angles in this configuration, the light shone onto the samples is reflected *away* from the camera, explaining the inability of the camera to detect any color from the films under this illumination condition.

Looking at the polarization, we see that the circular polarization is perfectly selected at normal incidence, the right-most sample appearing black through right-handed polarizer and the left and middle samples appearing black through the left-handed polarizer. All three appear at identical intensity without polarizer. At inclined incidence the polarization is not perfectly circular, since the eigenmodes are elliptical with non-zero eccentricity when light is incident at an angle to the helix^48,82^. Under diffuse light, the circular polarization contrast is still quite good, albeit not perfect, even at high *α*, whereas the polarization contrast is lost quite strongly, especially for the sample with red *λ*_0_, when the illumination is along −*α*. In addition, the color detected through the left-handed polarizer is different from that detected through the right-handed one. We speculate that this is due to the exceptional optical rotatory power of cholesterics, which is strongly wavelength-dependent in the vicinity of the reflection band^83^. This could change the orientation of the long axis of the elliptically polarized reflected light to varying degrees depending on the exact wavelength, leading to the color shift of the light passing through the circular polarizer.

Moving to the CSR samples, we note first that the well with near-IR CSRs appears almost white during imaging with collimated light. However, under ambient light and oblique incidence—which is how samples deployed in human-populated environments might appear to observers most of the time—the color of the sample with near-IR CSRs has an orange-red character. In fact, the reason for tuning *p* to near-IR retroreflection rather than red is the blueshifted cross communication and internal reflection signals (Fig. 1c), making it more challenging to produce CSRs that effectively appear red than CSRs that appear green or blue. While the well with CSRs with blue *λ*_0_ appears blue since the blueshifted signals are almost entirely in the invisible ultraviolet region, and the well with CSRs with green *λ*_0_ appears green with a blueish tint due to blue-shifted signals that are blue-violet, the combination of retroreflection and blueshifted signals for red *λ*_0_ spans the entire visible spectrum, even if the intensity varies depending on wavelength. By extending *λ*_0_^*air*^ to near-IR we hoped to achieve an effectively red color. To work under any illumination condition, *p* should have been even longer, but the partial success for ambient light illumination suggests that further refinement of the CSR design may yield CSR surfaces that appear red to the casual observer.

Importantly, all wells retain nearly constant color for all angles of observation under ambient light, in stark contrast to the drastic color change of the flat cholesteric films. The near-IR sample shows the greatest change for small inclinations, since it appears more whitish for *α* ≈ 0, but the red appearance develops already for *α* ≈ 10−15^°^. The green sample becomes slightly more blueish and the blue sample eventually gets violet as the observation angle increases, but overall we see a dramatically reduced color variation compared to the flat films. Also the polarization contrast is largely retained for ambient illumination.

In the second configuration, which maximizes the impact of the angle dependence of Bragg’s law, high *α* renders the green sample truly blue and triggers visible indiscriminate scattering in the blue sample. The latter is due primarily to the sample fraction of CSR beads with defects and imperfect alignment, which show up clearly as bright white stars on a dark background in this configuration. The well with near-IR CSRs appears quite strongly white, exhibiting a pinkish tone at intermediate observation and illumination angles. The polarization contrast is significantly worse in this configuration, in particular for the near-IR sample, where the ring-shaped internal reflections are visible regardless of polarizer (see Supplementary Fig. 3f).

**Fig. 4:**
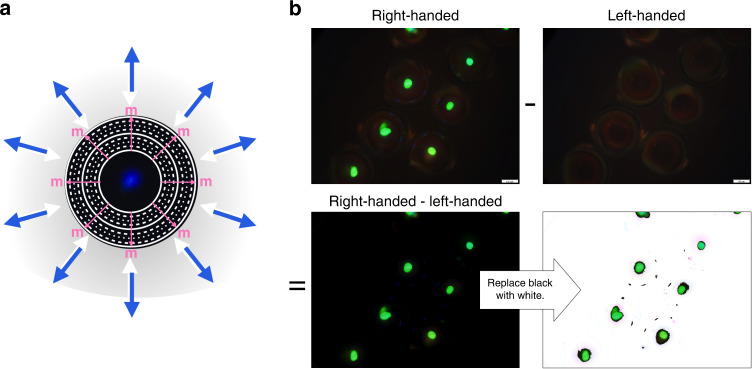
Chiral selective retroreflection of CSRs. **a** Schematic illustration of the omnidirectional selective retroreflection (white light in, blue light out, regardless of incoming light direction) due to the radial helix arrangement in a CSR shell with blue *λ*_0_. Pink arrows represent the local helix orientation **m**, white circles represent the director orientation (continuous: **n** in image plane; dotted: **n** perpendicular to image plane). **b** Illustration of polarization-based image subtraction, using reflection polarizing microscopy photos of a cluster of CSRs with green retroreflection. Scale bars: 100 µm

Where the CSR samples truly stand out is in the third configuration. Here there is practically no viewing angle dependence any more, neither for the intensity nor the color, and also the polarization contrast is quite good for the green and blue samples. Some scattering still reveals the samples in the wrong polarization channel, indicating the presence of many imperfections in these CSRs as well as an index-matching between CSRs and the NOA160 glue that is not perfect. The near-IR sample is again by far the worst, although its polarization contrast is acceptable at high incidence angles. Overall, this experiment shows that CSRs are true *omnidirectional selective retroreflectors*: they exhibit selective reflection in all directions, sending the selected component right back to the observer, see Fig. 4a.

Omnidirectional retroreflectivity is not unique to CSRs. Indeed, you find omnidirectional retroreflectors (often referred to just as ’retroreflectors’) on many road signs and in high-visibility clothing, as well as in the reflector balls used in motion capture work for research or entertainment. But in contrast to these standard retroreflectors, which reflect *all* light, making them strikingly visible^38^, the CSRs retroreflection is based on cholesteric Bragg diffraction, allowing us to select the wavelength band and the circular polarization that should be reflected. Because circular polarization is rare in nature (certain beetles exhibit circularly polarized reflection because their cuticle exhibits a cholesteric liquid crystal-derived structure^84,85^), the circular polarization of the selective CSR retroreflectivity is extremely powerful, as illustrated on microscopic scale in Fig. 4b. By subtracting a photo of an area containing right-handed CSRs taken through a left-handed polarizer from one taken through a right-handed polarizer, the unpolarized background is subtracted and only the CSR reflections appear in bright colors. By arranging the CSRs into specific patterns, such as alphabetical letters^29^ or QR-codes^28^, information can thus be inscribed using CSRs, and it can be read out with exceptional contrast since the background can be removed as illustrated. Moreover, since the CSRs are omnidirectional selective retroreflectors, the information can be read no matter from which direction the sample is illuminated, in contrast to the case if flat cholesterics were used, as clearly seen in the lower section of Fig. 3a.

Before demonstrating this potential in practice, let us briefly discuss the optical response also on the microscopic scale. The complete analysis is found in Supplementary Note 3; here we will only highlight one very interesting—and highly useful—aspect of the microscopic appearance of all the CSR samples. There is a significant uncontrolled variability of how the individual CSRs are organized within the binder, even when they are packed close to generate a macroscopically uniform appearance. Therefore, if CSRs are used to generate a well-defined and fully deterministic pattern as seen from a distance, an intrinsic randomness still arises at microscopic scale regarding the exact features of the individual CSRs making up the macroscopic pattern, see Supplementary Fig. 3d/j/p. When CSRs varying in size are mixed, the exact location of each CSR is entirely unpredictable, and if also more than one *λ*_0_ is used, there is an even greater individual variety at microscopic scale. Additionally, when shells are present, the orientation of their cylindrical symmetry axis may vary in an uncontrolled manner, as seen clearly in Supplementary Fig. 3d.

What this means is that every individual sample created by a sufficiently disperse mixture of CSRs carries a unique fingerprint that can be accessed by microscopic investigation, even if all samples generate the same macroscopic pattern. Arenas et al. showed that the identity space is large and that each CSR sample is sufficiently distinct that a certain sample can reliably be re-identified as original, while a different sample will be detected as non-original^41^. This is extremely powerful, because it means that patterns created using CSRs can be deterministic at macroscopic scale, in order to encode easily readable patterns like QR-codes onto an object surface, yet each individual copy of the pattern is unique and identifiable, allowing reliable authentication of the objects carrying the encoding. Furthermore, in part because it is practically impossible to reproduce the same arrangement of the same CSRs in a certain encoding, in part because the appearance depends strongly on the illumination and imaging conditions (Supplementary Note 4), one can argue that any array of CSRs with sufficient variability is a Physically Unclonable Function, or PUF^35,42^, allowing CSR coatings to provide very reliable authentication. The combination of unpredictable and deterministic features of CSR markers is a key feature for reliably linking physical objects to their digital twins, as we will discuss further towards the end of the paper.

### Encoding information onto surfaces using CSRs and detecting it with machine vision

Capitalizing on their unique optical properties described above, we see a significant potential of CSRs for encoding information to be read by machines that can detect the CSRs thanks to the polarization contrast, even if the CSRs are hidden in busy backgrounds. As we previously demonstrated CSR-encoding of text^29^ and QR-codes^28^, at the time staging the scene for taking images sequentially for different polarizations with a single camera and doing the post-processing manually in a time-consuming fashion, we here introduce hard- and software for dynamic real-time background subtraction, and we focus on a pattern that is particularly useful for robotics and AR, so-called *fiducial markers*. These are 2D binary markers, similar in design to QR-codes, that function as landmarks for robots and AR devices, supporting their navigation and operation in powerful ways^86,87^. By analyzing the pattern, size, rotation and perspective of a fiducial marker, a robot or AR device can easily and accurately identify the carrier of the marker and relate it to a digital twin/BIM, and then determine its own location, orientation and motion with respect to the item carrying the marker. But because traditional fiducial markers are highly visible, typically printed in black on a white background, they are rarely used outside research labs. A second challenge in using fiducial markers in arbitrary surroundings is false positives: many patterns in a complex environment like a modern cityscape might mistakenly be interpreted as a fiducial marker by a robot or AR device. By defining the marker pattern using CSRs, the risk of such false positives is removed since the background can be subtracted. And if the CRS reflections are located outside the human vision spectrum, there is a good chance to make CSR markers so difficult to notice by humans that they can be placed almost anywhere, without aesthetic impact on architecture and design.

#### Revealing CSR patterns with maximum contrast by using the CSR polarization to remove the background

We start by studying how patterns defined by CSRs can be detected and identified dynamically in real-time, even when they are placed in challenging environments. We focus particularly on elements such as windows and mirrors, since these are well known to cause significant problems for robots and AR devices when no fiducial markers are present. While the long-term aim is to design the markers for near-IR or near-ultraviolet (near-UV) operation, most cameras are fabricated with UV and IR blocking filters, and commonly available circular polarizers are optimized for the visual spectrum. For this reason, we demonstrate the real-time background subtraction and resulting pattern detection using a CSR marker designed for visible light operation. Specifically, it is made with right-handed CSRs with green *λ*_0_ embedded in UV-cured NOA160, and the areas with CSRs (corresponding to the black regions of traditional fiducial markers) have a black background coating. Regions without CSRs (white areas in traditional markers) consist of only NOA160 and have no background, hence they are fully transparent.

**Fig. 5:**
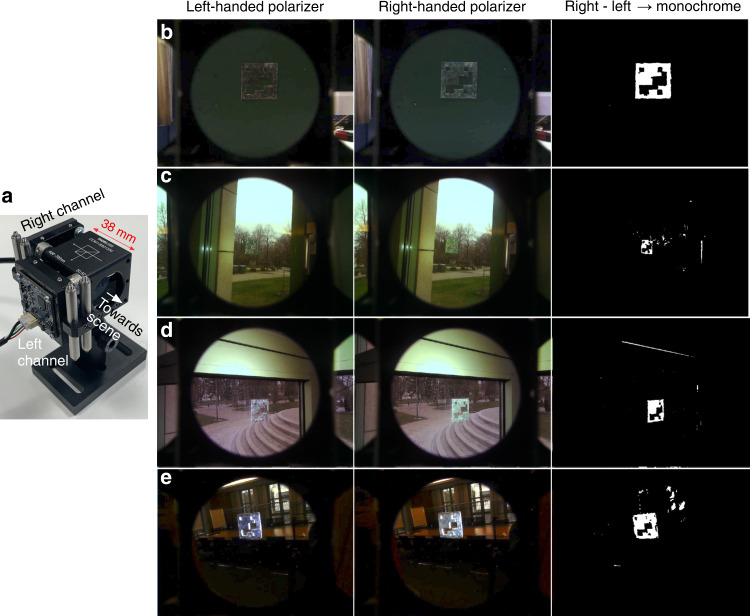
Detection of a CSR fiducial marker and removal of background using circular polarization. **a** Imaging set-up for background removal based on circular polarization, consisting of two cameras attached to a beam splitter, each camera having a right-handed circular polarizer in front of the lens. **b**–**e** Screenshots from software running real-time background subtraction and detection of a fiducial marker pattern (10 cm width) created by right-handed CSR shells (about 5–10% of beads are also present) with green retroreflection. The left and middle columns show the raw video feeds for left- and right-handed polarization, respectively, while the right column shows the subtraction of the left from the right channel, followed by switch to monochrome and post-processing. In panel (**b**), the marker is placed on a uniform metal plate painted with similar green color to that of the CSR retroreflection. In (**c**) and (**d**), the marker is placed on a window, with nature (**c**) and built environment (**d**) backgrounds, respectively. In (**e**) the marker is placed on a mirror, showing a room with windows reflected by the mirror. In all cases a white torch light placed next to the imaging set-up is illuminating the marker along the imaging direction. For clarity, the software screen shots in (**b**) and (**e**) have been adjusted digitally for higher exposure (equally over the entire area)

Our new dedicated read-out device (Fig. 5a) consists of a beam splitter, two circular polarizers and two standard USB cameras connected to a Raspberry Pi Linux computer. The circular polarizers, which are both right-handed, are placed directly in front of the lenses of the two cameras, which are mounted on two orthogonal sides of the beam splitter. The input aperture of the beam splitter is directed towards the scene to be analyzed, the semi-transparent mirror letting through half of the light along the straight path to camera 1, while the other half is reflected orthogonally to camera 2. Unpolarized components of the scene will appear identical to both cameras, so by subtracting the two camera images, these image components are removed. Circular-polarized components will hit the polarizer of camera 1 with the original circular polarization, whereas they will hit the polarizer of camera 2 with the inverted handedness, since a mirror reflection inverts circular polarization. This is the reason that both cameras have the same type of circular polarizer: a right-handed signal will be detected by camera 1 but not by camera 2, and vice versa for a left-handed signal.

The basic task of the software is to subtract the two video feeds in real time, switch to monochrome representation, and then present the output signal as the input to a standard fiducial marker interpretation algorithm. However, no two cameras are perfectly identical, and the physical mounting of the two cameras onto the beam splitter always leaves some mismatch between the fields of view of each. For this reason, the software first carries out an alignment of the two video feeds, detecting visual key points in corresponding frames from each camera and matching them using pixel-wise similarity analysis. Then the system performs the subtraction over the 2D image space and converts the resulting full-color image to monochrome. The system finally applies a post-processing step, filtering out outliers of unexpected sizes and locations. The software simultaneously shows the two raw video feeds and the resulting output next to each other, all feeds continuously updated in real time.

In Fig. 5b–e we show the performance of our system in detecting the CSR fiducial marker placed on three different surfaces, comparing a total of four scenarios. The first two columns show the raw video feeds of the two cameras and the third column shows the output signal from our software. In all cases the software runs continuously with real-time processing, and each row shows a single frame of the software output window. A commercial white LED torch is held next to the imaging set-up, oriented along the viewing direction to illuminate the marker, thereby ensuring retroreflection imaging conditions. The top row shows the marker on a calm uniform background, its color selected to be about the same green as the retroreflection color of the CSRs. In this way the marker is to some extent ’camouflaged’ to the naked eye, although several imperfections arising from the manual marker production (Supplementary Note 5) render it quite easy to detect. The software has no problem subtracting the feeds to highlight the CSR reflections, yielding the very high-contrast image of the marker pattern on the right in Fig. 5b.

Next, the marker is placed on the glass surface of a window, and the system is first oriented such that the sky and some trees and a lawn are seen through the window, see the two raw video frames in Fig. 5c. The alignment of the two video feeds in this case is not perfect, hence some noise remains in the output channel seen on the right. Nevertheless, it is clear that the marker is very well detected with excellent contrast. With further refinement of hard- and software, we expect the remaining noise to be removed. Figure 5d shows the marker on the same window, in a slightly different part of the glass, and the system is now oriented to image built environment (a stone staircase) through the window instead of the sky and nature scene of the previous example. The output stream again reveals the marker with excellent contrast, a slight mismatch in the video feed alignment producing a white line along one edge of the window frame. Again, this is due to imperfections in the hard- and software, hence it can safely be expected to be absent in the next generation of the system.

The final example, in Fig. 5e, shows the most challenging scenario, *combining* mirrors and windows. The marker is placed on the mirror in a room with several windows on the opposite side, thus appearing in the mirror image. The system still does a very good job in removing the background and revealing the marker with excellent contrast. As usual, imperfections in the alignment of the video feeds, this time aggravated by the complex lighting situation of small bright areas in an otherwise quite dark scene, leaves some spurious signals remaining. We can conclude that the principle of background subtraction based on the circular polarization of CSRs, and consequent identification of the pattern defined by CSRs, works very well even in real time, but further refinements of hard- and software will bring additional benefits.

#### Revealing CSR patterns with maximum contrast by using the retroreflection to remove the background

We recently started exploring an alternative approach to detecting CSR markers, designed in order to work with a standard smartphone without any additional hardware. Since current smartphones do not have circular polarizers, we here take advantage of the omnidirectional retroreflection of CSRs. The principle is simple: a smartphone camera takes two photos of a surface where a fiducial marker pattern has been encoded using CSRs, one with the flash turned on and the other without the flash, ensuring that the surface is inclined with its normal sufficiently away from the viewing direction. Since most surfaces are not omnidirectionally retroreflective, the flash light will primarily be reflected forwards, away from the phone camera, by the background surface. The CSRs, in contrast, reflect the flash light back to the camera regardless of imaging angle, since they are omnidirectional retroreflectors. A software would subtract the picture taken without flash from the picture taken with flash, which will have very similar fields of view even for a handheld phone if the software uses the high-speed video capability of modern smartphones. This should reveal the CSR pattern with high contrast.

**Fig. 6:**
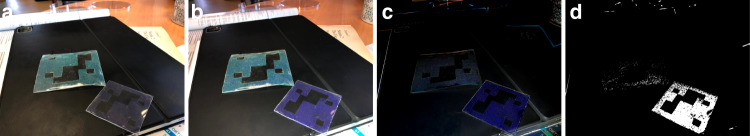
Detection of CSRs and removal of background using retroreflection. Two fiducial markers made using CSRs with green and violet retroreflection, respectively, photographed with a regular smart phone (iPhone X) at about 45^°^ angle with respect to the surface normal, using diffuse office ceiling lamp as sole illumination (**a**) and with the torch of the phone activated as well (**b**). In (**c**), the photo in (**a**) has been subtracted from that in (**b**). This is then separated into blue, green and red channels, after which the green channel is subtracted from the blue, following by a switch to monochrome mode with a threshold value of 15% to obtain (**d**). The image manipulations for obtaining (**c**) and (**d**) were done using Graphic Converter 9 (Lemkesoft)

We have so far tested the principle only with the regular camera software of the phone, taking the images manually with the flash turned on and off, respectively, and the images are then subtracted in a conventional graphics software. The result is shown in Fig. 6. A problem is that, even if the background surface is not designed to be retroreflective, it does scatter light, and some of the strong flash light is reflected back to the camera even at inclined viewing angles. Moreover, the regular camera software’s auto exposure and image processing algorithms are influenced by the flash light, leading to a non-negligible change in the background appearance. Nevertheless, the contrast between the CSR retroreflection and the background can still be good enough to use as intended. In the example, two CSR fiducial markers are placed on a matte black plastic surface, one marker being the same green-reflecting one as in Fig. 5, the other having violet *λ*_0_. The difference when the flash is turned on is immediately clear for the violet-reflecting fiducial marker, which has only a faint color in the other photo.

The green-reflecting marker is more visible in Fig. 6a than the violet-reflecting one, because the ambient light gives rise to blue-shifted reflections of nonretreflection character that are still in the visible range, reaching the camera even when there is no illumination along the viewing direction. Interestingly, when the flash is turned on, the green marker appears with enhanced blue color. Although *λ*_0_ is green, there are many internal shell retroreflection rings and cross communication signals excited that give a significant contribution. This means that, when (**a**) is subtracted from (**b**), the green marker appears in the output image (**c**) with rather strong blue tone, while the violet marker has the expected violet color. It is difficult to isolate the green marker, but the violet one can be extracted with good contrast by separating image (**c**) into the green, blue and red channels, subtracting the green from the blue, and then switching to monochrome, as shown in panel (**d**).

This mode of detecting fiducial markers is technically more challenging than the one using the circular polarization, but with dedicated software, both for the image subtraction and for controlling the camera and flash light, we believe it can work very well, at least for CSRs with blue-violet retroreflection. Since mobile phone cameras have UV and IR blocking filters, we cannot shift *λ*_0_ to near-UV in this application scenario. For CSRs with longer *λ*_0_, still in the visible range, the contrast can be enhanced by the aid of dyes introduced in the CSRs^62^.

#### Can the CSR marker concept be transferred to the near-UV and near-IR range?

By tuning *λ*_0_ to the near-IR or near-UV regions, one can envisage technologies in which code-generating patterns defined by CSRs are illuminated using light sources emitting in the corresponding parts of the spectra, invisible to human eye sight. Such technologies could be operated unobtrusively to humans, allowing them to be deployed in any environment. While any technology operating with UV light in the presence of humans and animals could be potentially dangerous, it is important to note that the distance into the UV range that we need to shift the operation wavelength to achieve invisible operation is very small. The reflection band width given by ∆*λ*^*LC*^ = *p*∆*n*^*nh*^ gets narrower the shorter the wavelength. Given a typical average refractive index $\bar n = 1.6$ and assuming that any air wavelength greater than *λ*^*air*^ = 380 nm could be visible to humans, we would need to ensure that the reflection band, as measured in the CSR, stays shorter than *λ*^*LC*^ = 380*/*1.6 = 237.5 nm. Assuming ∆*n*^*nh*^ = 0.1, we see that a CSR with *p* = 225 nm would work very well, as its long-wavelength end of the reflection band would be (in the CSR) 225 + 22.5*/*2 = 236.25 nm, which in air corresponds to 378 nm. The air wavelength for operation would then be *λ*_0_ = 225·1.6 = 360 nm, which is nearly identical to the UV component emitted by ordinary compact fluorescent lamps, which exhibit significant emission at 365 nm^88^. Also ordinary sunlight at earth level has high intensity of light in this wavelength range^89^. This means that operation at these near-UV wavelengths can be considered perfectly safe. A problem of more practical nature is that many standard near-UV emitters on the market today, so-called ’black-light’ lamps, also emit visible violet light, hence such illuminators give away the technology. One would thus need a very narrow-band near-UV emitter for illumination, probably with a blocking filter that removes visible as well as any light unnecessarily far into the UV.

**Fig. 7:**
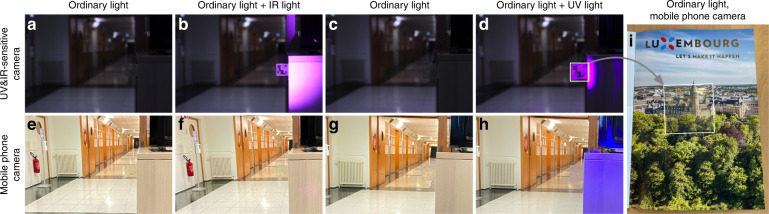
Near-IR and near-UV-reflecting fiducial markers in indoor environment. Macroscopic views of fiducial markers made of CSRs with near-IR (columns 1–2) and near-UV (columns 3–4) retroreflection, respectively, in NOA160, as seen by a modified DSLR camera without UV/IR blocking filter (top row) and by a regular mobile phone camera (bottom row). The markers are illuminated by the regular white ceiling light in (**a**/**e**) and (**c**/**g**), additionally by a 940 nm night vision LED in (**b**/**f**) and by a black light lamp in (**d**/**h**). While the pattern of each marker is difficult to see under ordinary light illumination only, it becomes very clear with the IR/UV-imaging camera when the corresponding near-IR/UV light is turned on. Note that no polarization filters are used, hence the background is not subtracted. The near-UV marker placed on a printed magazine page is photographed using a regular mobile phone camera in (**i**), showing that it is nearly fully transparent and difficult to notice by the naked eye (as a guide to the eye, a white frame highlights its location). The few scattering dots are CSR beads rather than shells. The outer side length of each marker is 5 cm

For near-UV CSRs, the blue-shifted reflections due to cross communication and internal shell reflection cause no problems, since they occur for wavelengths even further from the visible spectrum. Indeed, already the blue-reflecting CSRs in Fig. 3 demonstrate this advantage: beyond the central retroreflection spots, they reflect no visible light. A second significant advantage is that visible light cannot distinguish the very short-pitch helical modulation within near-UV CSRs. While the anisotropy makes index matching challenging (see Supplementary Note 6), there is no visible light scattering *within* the CSRs, and we will soon see (Fig. 7) that good enough index matching can be achieved to render near-UV CSRs very difficult to detect by eye^29^.

For near-IR operation, there are two challenges to take into account if the CSRs should not be detectable by human eye sight. First, the blue-shifted signals from photonic cross communication and shell-internal retroreflection, as well as the blue-shifted forward reflections when the light source is opposite to the observer as in the second imaging configuration in Fig. 3b, will all contribute to giving away the existence of the CSRs to human observers if *λ*_0_ is not long enough. Fortunately, the refraction at the interface between air and the binder surrounding the CSRs limits the maximum Bragg angle θ, which compares the light propagation direction within the cholesteric to the orientation of the helix axis, and one can thus estimate that no visible reflections will occur if *λ*_0_ > 1.7 *µ*m^29^. A technology operating at such long wavelength would be costly, since standard consumer IR technology, like night vision equipment, operates at 850 or 940 nm. Equipment operating at wavelengths greater than 1 *µ*m is significantly more expensive. Interestingly, one may now *take advantage* of the blue shift due to photonic cross communication and shell internal retroreflection, designing the technology not for operation at *λ*_0_ but for *λ*_0_ cos〈θ〉, where 〈θ〉 is a representative average angle corresponding to the most prominent signal. Geng et al. demonstrated that CSRs with *λ*_0_ = 1.9 *µ*m can be very well detected with standard night vision equipment based on this principle^29^.

The second challenge is that near-IR CSRs scatter visible light in themselves^29^. This is because, in contrast to the case of near-UV CSRs, visible light detects the modulation along the helix, resulting in non-selective scattering. This makes it impossible to make near-IR CSRs completely invisible to the human eye, thus limiting the contexts where a CSR technology designed for near-IR operation could be applied. However, there are many surfaces in our environments that scatter light in themselves, and these surfaces may be suitable backgrounds for near-IR CSRs.

In order to realize the fiducial marker detection and background subtraction demonstrated in Fig. 5b–e when using CSRs that have been designed to be invisible to humans by shifting *λ*_0_ to the near-IR or near-UV range, we are in the process of rebuilding the set-up in Fig. 5a with tailored equipment. While circular polarizer films for the visible range are mass-produced for use in 3D cinema goggles and thus readily available for incorporation in the set-up that we used for visible operation, the corresponding circular polarizers for UV and IR operation are rare to find and their design leaves less flexibility in incorporating in a portable set-up based on a small beam splitter. Likewise, standard cameras cannot detect light outside the visible spectrum due to their integrated UV/IR blocking filters, hence they cannot be used.

To test at least the retroreflection of the CSRs in the near-UV and near-IR ranges, we use a modified DSLR camera in which the UV/IR blocking filter has been removed (modification by Llewellyn Data Processing LLC), and we use standard commercial LED lighting for night vision and for ’black light’, respectively, to illuminate the scene, see Fig. 7. In the first two columns, a fiducial marker made with CSRs designed for near-IR operation is placed in an indoor environment, placed without direct background protruding from a cabinet. In panel (a) it is imaged with the modified camera without the IR light illumination whereas in (b) this light is turned on (operating at 940 nm night vision wavelength). Note that any IR or UV reflections appear purple in the image because the camera was designed for regular visible photography, and after removing the IR/UV blocking filters, either category of non-visible light triggers the same purple signal. The images in the lower row are taken under the same conditions, but with a regular mobile phone camera where the IR/UV blocking filter is intact.

Without IR light, the marker and its pattern are somewhat visible in Fig. 7a, because *λ*_0_ is too close to the visible. Nevertheless, the visibility is quite low and the marker appears largely transparent, as seen in particular in panel (e), taken with an ordinary mobile phone camera. Also in (f), taken with the mobile phone camera when the IR light is on, the marker visibility remains low. In fact, there is no detectable difference between (e) and (f), because the IR illumination source has no visible component. In stark contrast, the marker pattern appears with great clarity in panel (b), taken with the modified camera when the IR light is on. The entire square of the marker is filled with binder (cured NOA 160), but only the regions with CSRs appear with the purple color with which the camera represents IR light. This shows that, indeed, the selective retroreflectivity of CSRs works also in the near-IR range, including standard night vision wavelengths.

We do the same experiment with a fiducial marker made using CSRs designed for near-UV retroreflection in Fig. 7c–d/g–h. This time it is important to note that the black light illuminator emits also in the visible violet range, making the illumination visible also to the regular mobile phone camera, as seen in panel (h). The fiducial marker is colorless this time, as expected, but some scattering still reveals the marker pattern to the eye under ordinary illumination upon close inspection, see panels (c) and (g). When placed directly on a background, as in panel (i), this scattering is much less prominent than in the freely suspended configuration, hence the near-UV marker can indeed be made very difficult to detect by eye.

When the blacklight is turned on, the CSR-defined pattern appears strongly in the image taken with the modified camera (Fig. 7d), again with no reflection from the NOA160 regions without CSRs. This confirms the excellent retroreflection behavior in the near-UV range when *p* is tuned accordingly. This time, the pattern appears slightly stronger also in the image taken with the regular mobile phone camera (h), which is due to the violet component of the flood light; these CSRs have *λ*_0_ so close to the violet edge of the visible spectrum that the reflection band extends slightly into the visible.

### Application opportunities of CSR markers

The possibilities of exploiting the peculiar properties of CSR markers—from the ease in detecting pre-defined patterns over a distance without false positives thanks to the polarization- and wavelength-selective retroreflectivity to the unique unclonable PUF identity revealed by investigating a cluster of CSRs at close distance—are limited only by imagination. The next step is to make the communities that could profit from technologies based on CSR markers aware of the possibilities, in order that further application opportunities are identified. This will, in turn, motivate efforts to solve the remaining engineering challenges related to upscaling and automation of fiducial marker production, as well as adaptation of machine vision systems to detect human-invisible CSR-encoded information. We end the article by describing some application opportunities that we are currently working towards, hoping that these examples will inspire further exploration.

#### Supporting Augmented Reality (AR) and Simultaneous Localization and Mapping (SLAM) of robots using CSR-based fiducial markers that are undetectable by the human eye

The reason that fiducial markers are popular in robotics and AR research, as well as in certain restricted facilities like nuclear power plants or military installations, is that they greatly simplify the computational tasks for camera pose estimation and related simultaneous localization and mapping (SLAM) and path planning^86,87^, as well as the manipulation of objects carrying the markers. Adding the fact that the library of fiducial marker patterns can be used to classify the objects carrying the markers, even if they look identical apart from the marker, it becomes easier for robots and AR devices to make sense of their environment. For instance, doors that all appear identical can have different markers that tell a robot what is behind each door.

While existing fiducial marker libraries have limited identity space, there are many possibilities of expanding them in smart ways when the markers are made using CSRs. For instance, the same marker pattern could be realized using near-IR and near-UV CSRs, with right- as well as left-handed polarization, to distinguish different versions of an object for different categories. One could also reserve a certain wavelength range and polarization for specific users, for instance near-IR left-handed for commercial use, near-IR right-handed for individual use, near-UV left-handed for state authority use, and near-UV right-handed for military use. That way, multiple human-invisible yet highly information-rich landscapes can be created in parallel, and a device interested only in one category will remain undisturbed by the others.

The increased reliability of robot performance when fiducial markers are available to support SLAM means that an infrastructure of CSR fiducial markers could increase trust in all kinds of robots, from self-driving cars to drones and personal assistance robots, facilitating their deployment in human-populated environments. In the future, we may imagine CSR markers operating outside the visible spectrum placed on key components of typical city traffic scenes, greatly increasing the reliability of self-driving cars by their improved ability to analyze the scene. A few tragic accidents involving self-driving cars during the last years have slowed down the roll-out of this technology, hence any technology that can significantly reduce the risk of such accidents may have a positive impact. Note that the markers would not violate personal integrity, because they would only tell that a certain component is a tram, a cyclist or a car, they should say nothing about the individuals in the scene. Following this principle, CSR-based fiducial markers could be worn by humans to minimize the risk of accidents caused by any type of robot they encounter; the marker would simply represent the category of ’this is a human’, without any further identification. Because the computational requirements for analyzing the environment goes down when fiducial markers are deployed, robots and AR devices would consume less energy and they could be designed with less advanced image processing capacity. In particular, machine vision powered by artificial intelligence (AI), capable of face recognition, would in many cases become unnecessary and might even be actively blocked to minimize privacy-intrusive effects of the machine deployment among humans^90^.

#### Creating trustworthy links between physical objects and their digital twins, and thereby fighting counterfeiting, using CSR markers

In contrast to the situation for human-worn CSR markers, there is good reason to use the full power of CSR markers on physical objects to identify them uniquely, for instance to increase supply chain transparency and traceability, and to identify originals from fakes in markets rife with counterfeits. For such application, each CSR marker would have its microscopic PUF characteristics enrolled and securely stored as part of the digital twin of the object carrying the marker. Robots, AR devices or other equipment handling the objects could read these data during close-up investigation to establish a reliable and trustworthy link between the digital identity and the physical counterpart. Meanwhile, they would read the deterministic marker pattern from afar, using a set-up like that in Fig. 5, to categorize items and establish their locations and orientations with respect to themselves.

This concept can be applied to almost any digital twin model, forming a perfect complement to many blockchain solutions which secure the digital identity but do not give a unique link to the corresponding physical item. There is a very strong trend today of moving towards blockchain and digital twins for track-and-trace tasks to secure supply chains, but the vulnerability lies in ensuring that a physical object truly corresponds to the digital twin. Currently, the physical object is typically identified with easily copiable QR-codes, sometimes with RFID chips, most of which are also easy to clone. Since we can make fiducial markers using CSRs, we can make many other types of graphical marker, including QR-codes, bar codes or serial numbers, using CSRs, unleashing many advantages. First, for products that are prone to counterfeiting, such as pharmaceuticals, electronics or jewelry, the unclonability of the marker offers an enormous advantage over current serialization techniques. Second, if the code can be hidden from human vision it can be made as large as the packaging. This, together with the ease of machine vision systems in detecting it without false positives as described above, can allow logistics robots and other devices to monitor stock-keeping units with high fidelity even over large distances.

Even for consumers, CSR markers could be very useful. As demonstrated in Fig. 6, the technology of current smartphone generations is already sufficient to isolate and read CSR markers. With dedicated software, read-out should be possible by simply comparing images taken with and without flash. A consumer could use this to verify the authenticity of products on supermarket shelves where fakes are common, for instance expensive wines or other alcoholic beverages. Interestingly, future generations of smartphones may well be equipped with image sensors that are able to detect polarization directly^91^, and then every smartphone will be able to read CSR markers extremely efficiently and reliably, as also the background subtraction demonstrated in Fig. 5 could be used without additional hardware. While integrated IR/UV blocking filters will most likely require the CSRs to operate in the visible range, they may be well hidden by arranging them following a camouflage-like principle. Allowing consumers to read the CSR-based markers when they so wish is important also for acceptance of the new technology.

#### CSR markers in construction of the built environment and circular economy solutions

An area where we believe the adoption of a technology based on CSR fiducial markers is particularly promising is the construction of the built environment, one of the most lucrative industries in the world^92^. Especially in combination with the deployment of robots assisting the construction workers, CSR fiducial markers on surfaces of construction elements, as well as machines and workers, could be highly beneficial. Since many modern construction sites already adopt BIM or similar digital twin models for planning the construction, the digital counterpart is already in place. With invisible uncloneable CSR-based fiducial markers on components in a construction site, not only will the use of robots in construction be greatly facilitated, removing tasks that have a particularly high toll on human workers (e.g. tiling), but each physical item of the building now acquires a reliable link to its digital twin computer model. If the markers are successfully hidden from human eyesight, they can be deployed anywhere on any component, even in those ending up on a building facade. In cases where the surface is covered by paint or by other components, the invisibility is not important from an aesthetic point of view, but it may still be desirable as it renders sabotage of the system by removal or obstruction of markers more difficult.

The value in a reliable linking of construction elements to the digital twin is well recognized, and on-going efforts explore solutions using primarily IoT or RFID devices to perform this function. While such devices are extremely useful, their incorporation can be challenging and costly, and IoT devices require continuous power supply. If a passive and unclonable link can be realized at low cost, this will be a highly valuable complement, for instance when the key goal is to maintain trustworthy data on the quality of a certain type of steel, concrete or wood used in the construction. As the world is increasingly looking for sustainable solutions, in particular those of the circular economy, reusable construction components are becoming more and more interesting^93^. Maintaining the data about such components, over long time frames, then becomes even more important. To assess whether a certain reusable element is suitable for a new type of usage, for instance in a new building erected using reusable elements salvaged from a building that has reached its end of life, it is vital that the digital twin contains absolutely reliable data on each individual element of the old building. Here CSR markers could be very powerful, as their non-reliance on power supply, unclonability and unique identity allows each reusable element to be identified and linked to its digital twin. In this case, the demands on the materials used for the marker in terms of mechanical resistance and lifetime of years to decades (depending on the types of construction) become significant, but based on performance of existing advanced polymeric coatings, we are optimistic that this can be ensured. There is a generally increasing demand for materials traceability in industrial production, again often involving a digital twin for storing a ’materials passport’ for every product. CSR markers have enormous potential to facilitate such developments.

Beyond the linking function based on unique and unclonable marker identity, also the general marker pattern on construction elements is very powerful for enabling robotic assistance at a construction site. By definition, a construction site constantly changes its appearance, which is highly challenging for robotic localization and navigation. If every element carries fiducial markers, especially if they are made with CSRs, enabling efficient background subtraction and detection without false positives, robots on the construction site can continuously adapt to the changes as the edifice is gradually erected, since it needs only read the marker patterns to update its model of the site. If the workers also wear the CSR markers on their helmets and uniforms (without identifying individuals), any machine on the site, a robot, crane or other machine that could kill a person that unannounced enters its path of operation, would easily and reliably detect a person in the wrong place, and it could be programmed to then carry out an immediate emergency stop automatically.

These are just snapshots of potential application scenarios of CSRs used to encode machine-readable information, focusing particularly on their appearance in graphical markers that allow machines to quickly categorize and localize items they interact with, in addition to reliably authenticating items with respect to their unique identity when desired. Some scenarios may turn out not to be competitive, but many more will show up when the technology has matured further. We are convinced that the unique photonics of CSRs can be used to solve a range of problems, not least in addressing issues with emerging technologies where humans and machines find themselves coexisting in environments very different from factory floors or research labs. There are still many challenges to take on, from chemistry optimization to in-depth elucidation of optics to hardware redesign and software development, but the potential gains are so high that we are certain that all efforts to address these challenges will pay off.

## Supplementary information

Supplementary information

## Data Availability

All raw data supporting the findings of this study will be made openly available upon acceptance in a repository at zenodo.org.
