# Supplementary material for: Unclonable human-invisible machine vision markers leveraging the omnidirectional chiral Bragg diffraction of cholesteric spherical reflectors
Source: Light Sci Appl. 2022 Oct 25;11:309. doi: 10.1038/s41377-022-01002-4 (PMC9592545; doi:10.1038/s41377-022-01002-4)
Supplement: Supplementary file 1 — Supplementary information [file 41377_2022_1002_MOESM1_ESM.pdf]

Unclonable human-invisible machine vision markers leveraging the omnidirectional chiral Bragg diffraction of cholesteric spherical reflectors

Hakam Agha[a+], Yong Geng[a+], Xu Ma[a], Deniz Isinsu Avsar[a], Rijeesh Kizhakidathazhath[a], Yan-Song Zhang[a], Ali Tourani[b], Hriday Bavle[b], Jose-Luis Sanchez-Lopez[b], Holger Voos[b], Mathew Schwartz[c], and Jan P.F. Lagerwall[a*]

[a]University of Luxembourg, Department of Physics & Materials Science, 1511 Luxembourg, Luxembourg
[b]University of Luxembourg, Interdisciplinary Centre for Security, Reliability and Trust (SnT), 1855 Luxembourg, Luxembourg
[c] New Jersey Institute of Technology, College of Architecture and Design, University Heights, Newark, NJ, USA
[*]Jan.Lagerwall@lcsoftmatter.com (J.L.)
[+]These authors contributed equally to this work

SUPPLEMENTARY INFORMATION

Supplementary Note 1: Molecules forming cholesteric liquid crystals that can be polymerized into a solid state

As with any thermotropic (temperature-controlled) liquid crystal phase, the cholesteric phase is thermodynamically stable only within a limited temperature range. In order to ensure that this range is large enough and that it includes the temperature window within which the cholesteric is processed, we always work with multi-component mixtures, ideally with a near-eutectic composition. The composition of a mixture that we have used for many of the samples in this paper is shown in Supplementary Fig. 1. The approach also allows us to easily tune the helix pitch and select the handedness, because most often the mixture consists primarily of non-chiral liquid crystal-forming molecules (mesogens) and only a small mole fraction x of a chiral dopant, the sole function of which is to impose the helical modulation of the director. Its ability to do so is called the Helical Twisting Power (HTP), and it is defined as $\lim_{x\to 0} \frac{\partial q}{\partial x}$, where we have introduced the helical wave vector $q = 2\pi/p$, which can be seen as a convenient measure of the strength of twisting of the director in the cholesteric. The HTP depends both on the chiral dopant and host, but generally a dopant with high HTP in one host will also exhibit high HTP for another, albeit with a slightly different value. Ideally, the chiral dopants used to make CSRs have so high HTP that a concentration of 1–2 mol-% is enough to bring p into the target range, since this means that the variation of x required to tune p where we want it has negligible impact on the temperature range of the cholesteric phase. By using the opposite enantiomer of the chiral dopant at the same concentration x, the same p is obtained but with opposite handedness.

Since we will need to make the cholesteric liquid crystal-derived self-organized order permanent, we work with reactive mesogens, i.e., liquid crystal forming molecules that contain at least one moiety that can take part in a polymerization reaction. The most common choice is the acrylate group, CH_2=CHCOOR, where R is the rest that represents the mesogenic part of the molecule. Reactive mesogens typically come with one or two acrylate groups (compound **1** in Supplementary Fig. 1 has two), most often at the end(s), although they can also be attached to a side chain extending from an intermediate part of the

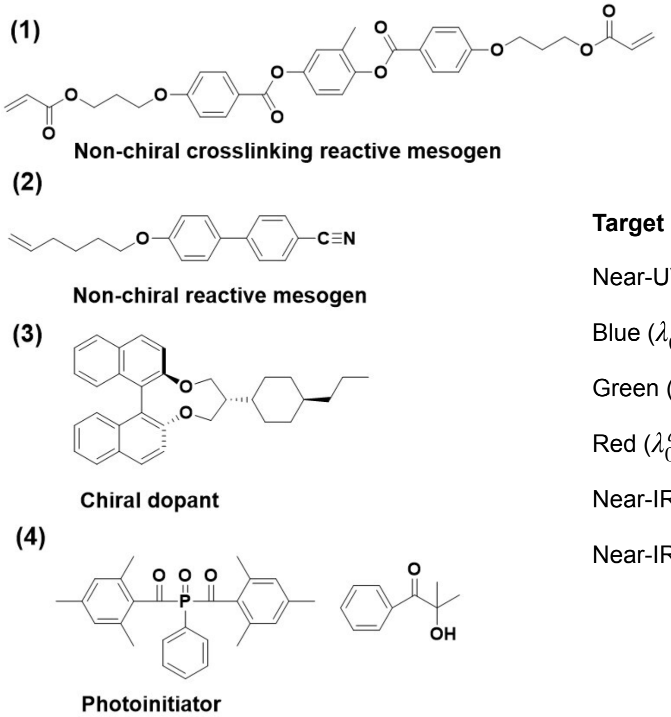

Target operation range	$x_{(1)}$ [mol-%]	$x_{(3)}$ [mol-%]
Near-UV ($\lambda_0^{air} \approx 385$ nm)	21.8	2.5
Blue ($\lambda_0^{air} = 458$ nm)	22.1	2.2
Green ($\lambda_0^{air} = 554$ nm)	22.4	1.9
Red ($\lambda_0^{air} = 690$ nm)	22.7	1.6
Near-IR 1 ($\lambda_0^{air} \approx 0.88$ µm)	23.0	1.3
Near-IR 2 ($\lambda_0^{air} \approx 1.1$ µm)	23.5	0.8

Supplementary Fig. 1. Chemical constituents of a suitable cholesteric precursor mixture for CSR production. To make a reactive non-chiral nematic base mixture we combine a bifunctional mesogen (1) with a monofunctional mesogen (2), the former acting as a crosslinker to ensure that a rigid polymer is formed upon initiation, the latter added to lower the melting temperature. The chiral dopant (3) is added at a concentration selected as to tune the pitch to the desired range, and the photoinitiator (4)—here a mixture of two components—is added to allow initiation of polymerization on demand via UV-irradiation. For simplicity, we kept the concentrations of (2) and (4) constant at 75.7-mol-% and 2 mol-%, respectively, varying the concentrations of (1) and (3) as indicated on the right to tune λ_0 in the final CSRs, after polymerization, to the targeted range.

molecule. Some reactive mesogens, like compound **2** in Supplementary Fig. 1, have only a carbon-carbon double bond rather than an acrylate group as reactive moiety. They can be used as well, although the efficiency of polymerization is often lower for such molecules, which can be a problem. To choose when the polymerization takes place, a photoinitiator is typically added to the cholesteric mixture, in some cases also an inhibitor to ensure that no premature polymerization takes place. The photoinitiator is sensitive mainly to UV light exposure, but also blue light can trigger initiation, hence all processing until the time of polymerization is done in labs with yellow light. Once the desired cholesteric structure is confirmed, the sample is exposed to UV light in a controlled way, turning the photoinitiator molecules into free radicals that start the polymerization and crosslinking of reactive mesogens into a solid network. Especially if the cholesteric is well confined during this process, and the polymerization is fast enough, the self-organized cholesteric helix can remain intact to a high degree, thus transferring the cholesteric optical properties to the produced solid.

A challenge when tuning the cholesteric mixture for a specific λ_0 is that the CSR production method (see below) comprises two steps during which p shrinks, and possibly a final expansion step in addition.[1] First, the polymerization leads to shrinkage and, second, an acetone washing step to remove unreacted components and to finalize shell puncturing typically leads to additional shrinkage after the acetone has been removed. If the binder swells the CSR to some extent, then p will expand. If any aspect of the CSR production procedure is changed, the overall variations of p need to re-established empirically, rendering such modifications somewhat tedious.

The composition of the liquid crystal mixture is important for ensuring the appropriate helix pitch, viscosity and temperature range of the cholesteric phase, but it also strongly influences the behavior and life time of the liquid state shells after production. First, the permeability of the immiscible isotropic solvent surrounding the LC (most often water) can be quite different for different mixtures, with significant impact on the ability to speed up annealing using osmotic stretching. Second, and also related to the first point, the stability of the liquid state shell turns out to be strongly dependent on the mixture components, at least when tangential alignment is required, as in the case of CSRs. While surfactants can be very powerful in stabilizing shells, they also tend to induce normal director alignment (although this also depends on concentration and type of surfactant[2]), which is the opposite of what we need for CSRs. In a recent study of shell-shaped liquid crystal elastomer (LCE) actuators,[3] we found that shells made using a reactive mesogen that is very popular for LCEs were impossible to stabilize with tangential alignment if no other mesogens are added. Noting that almost all work on liquid crystal shells has been made using the classic cyanobiphenyl-based class of mesogens (common choices being the single-component compounds 5CB and 8CB and the quarternary mixture E7), we added 1 mol-% of a cyanobiphenyl mesogen, finding a remarkable stabilization of the shells that allowed sufficient lifetime for annealing followed by polymerization into LCEs. We see the same effect with CSR mixtures: if the mixture contains no cyanobiphenyl compound, the risk of shell rupture and droplet fusion is much greater than when such a component is present. We thus make sure to include at least 1 mol-% of a cyanobiphenyl-based mesogen in all mixtures. It appears that its slightly amphiphilic character in combination with its mesogenicity has a highly beneficial impact on the stability of the shell, ensuring the lifetime required for annealing prior to polymerization. Once the shell has been polymerized, stability is no longer an issue, since the liquid state is now replaced by a solid one.

Supplementary Note 2: Molding cholesterics into spheres

One of the most exotic features of liquid crystals is that they remain liquids despite their long-range ordered structures. The latter is a feature we otherwise see only in solid crystals. The liquid state of cholesterics is key to making CSRs, since it allows us to process the cholesteric like any other liquid, molding it into different shapes. To make CSRs, we use standard microfluidic or other emulsification tools to prepare spherical droplets or shells of cholesteric, the size being tunable in the range from a few microns (for droplets) to a few tenths of millimeters (if uniform alignment is required, such large sizes are achievable only with shells). A CSR shell is typically made using a nested capillary microfluidic pathway,[4,5] in which an inner aqueous phase is injected into a cholesteric liquid crystal that flows in the same direction, but this meets another counter-flowing aqueous phase that, by virtue of its immiscibility with the cholesteric, flow focuses it into a collection tube, see Supplementary Fig. 2a. Until recently, this has been a highly manual procedure using hand-made devices, but at least one commercial device (Secoya Raydrop) for high-volume shell production is now on the market. This flows all three phases (inner, shell phase and outer phase) in the same direction, through a 3D-printed nozzle, which produces the shells with high reliability and reproducibility. With this procedure, shells with diameter ranging from about 100 μm to several hundred μm can be produced, the average thickness often being on the order of 3–10 μm. If smaller diameter shells are needed, a modification of the basic microfluidic design referred to as tip streaming[6] can be used, although it has not yet (to the best of our knowledge) been demonstrated with cholesteric liquid crystals. While the production itself is intrinsically serial, the production speed can be high (10–100 shells per second) and by combining multiple production units in parallel, the yield can be dramatically scaled up.

It is critically important that the helix aligns radially and for this reason the stabilizers and isotropic phases are selected such that they promote director alignment that is tangential to the interface, since $\mathbf{m} \perp \mathbf{n}$. Conveniently, an excellent choice for the solvent of the surrounding isotropic phases is water, as the key requirement is that these phases are immiscible with the

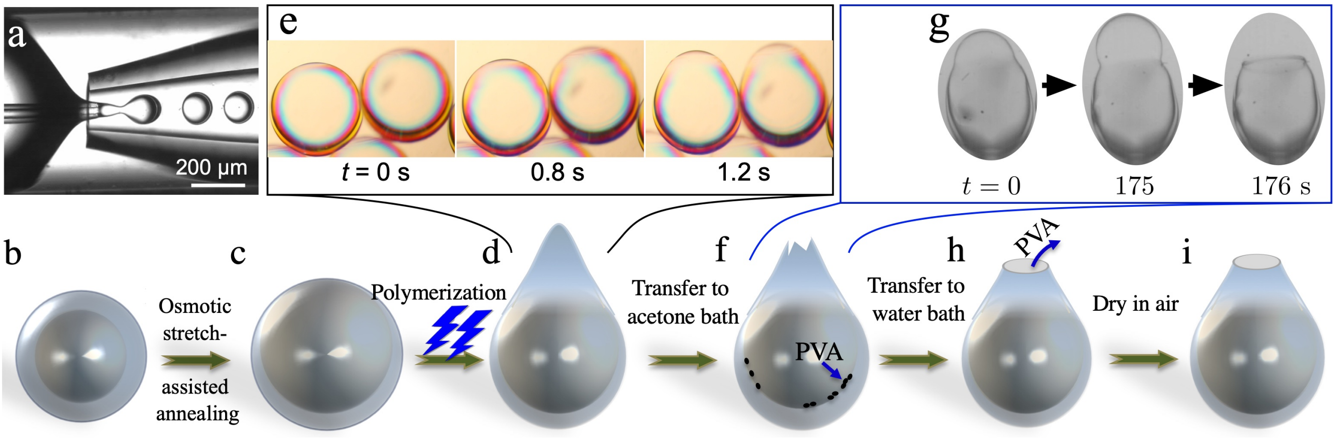

Supplementary Fig. 2. CSR shell production. a) Microscopy from production. **b–i)** Schematic with the full production process; **e)** Microscopic side views of asymmetric shells bulging out at the thinnest point due to polymerization-induced shrinkage. **g)** Shell bursting at the protruded point during washing with acetone. Panels (b)–(d) and (f)–(i) are reproduced from[1] (CC BY 4.0).

liquid crystal and have suitable density and viscosity. There are many suitable water-soluble stabilizers, typically polymers or surfactants, with which we can prevent collapse of shells or merging of droplets, while at the same time controlling the alignment of the director at the boundary of the cholesteric phase. We frequently work with polyvinylalcohol (PVA), a commonly used low-cost polymer with small environmental footprint that is produced at industrial scale.

It is by propagating the order imposed by the boundary conditions into the bulk of the liquid crystal that a uniform radial helix alignment is ensured, but this can take some time, in particular if the cholesteric mixture is viscous. Therefore, a stage of annealing is often introduced between production of the liquid CSR and the polymerization into a solid. With CSR shells, the annealing time can be significantly reduced by osmotic stretching of the shell, as already mentioned. The osmotic stretching corresponds to a compression of the shell thickness along the shell radius. Such anisotropic compression promotes helix alignment along the compression direction, in the same way as 'anisotropic deswelling' is used to align cholesteric liquid crystal elastomers or cholesteric colloidal suspensions.[7–9] In our 2016 paper where this approach to speeding up CSR annealing was first presented,[5] we reduced the annealing time from several days to about 12 hours, using an aqueous solution of PVA inside and pure water outside. The details of the process depend on many parameters, and we are in the process of fine-tuning these to reach very short annealing times. Currently we have been able to reduce the annealing time down to six minutes for a fully optimized situation. Such speeding-up of the annealing is very important for the prospects of scaling up CSR production, since it will enable continuous flow processing of all the steps in Supplementary Fig. 2a–i, from injection of the precursor mixture to collection of polymerized CSRs.

Another important feature of CSR shells is the above-mentioned feature that they generally end up asymmetric due to the density mismatch between the liquid crystal and the inner phase. When polymerizing such a shell, this asymmetry has important consequences, because polymerization leads to shrinkage of the cholesteric liquid crystal, yet it forms a self-closing shell around an incompressible aqueous phase, which is not shrinking in the process. The stress build-up thus leads to a deformation from a perfect spherical shape, where the thinnest and thus weakest point bulges out to let the (now too large) inner droplet protrude somewhat,[1] see Supplementary Fig. 2e. In some cases, the deformation is so strong that the shell breaks, but in most cases it remains intact but deformed after polymerization. However, if we soak the polymerized shells in an acetone bath (Supplementary Fig. 2f), the swelling stress that results, very different in different parts of the shell due to its asymmetric thickness and deformed ground state, does lead to breakage of the shell near the thinnest point. We thus get a single hole in each shell, where the thinnest point was, while the majority of the shell retains its well-defined spherical shape since the crosslinked polymer network prevents it from collapsing.

It is much easier to make CSR droplets than shells, and it is quite easy to reach small sizes around 1–10 μm diameter, but droplets have the problems related to the optical properties that we have discussed in detail in the main paper. The easiest way to make CSR droplets is to stir, shake or sonicate (treat with ultrasound) a container in which the cholesteric mixture is contained together with an immiscible isotropic liquid,[10,11] possibly a solution of a suitable stabilizer. This mechanically separates the liquid crystal into many small droplets, which under the influence of interfacial tension adopt a spherical shape. The stabilizers ensure that the interfacial tension is not too high and that the droplets do not coalesce if they come into contact, thereby making the system stable over long enough time to process the CSRs. The droplets produced in this way are, however,

highly disperse in diameter, hence if a single-size CSR is desired (in most cases this is not the case), microfluidic methods are required. With such methods, it is relatively easy to produce non-disperse droplets with diameter on the order of 30 μm repeatedly and at high throughput.

An interesting combination of CSR droplet and shell was devised by the Kim group.[12] A non-reactive cholesteric droplet is emulsified within a layer of cholesteric liquid crystal containing reactive molecules, which are crosslinked after production. This means that two different concentric cholesteric structures are present, one permanent and one dynamic. The same group also explored another way of making cholesteric shells that yields multiple concentric shells.[13] In this case, a droplet was produced using a microfluidic set-up but its composition was such that it becomes unstable once one component leaves into the continuous phase after production. As a result, phase separation within the droplet occurs, eventually stabilizing single or multiple concentric cholesteric shells. The procedure is very elegant and the resulting structures fascinating. Whether they are useful for applications remains to be seen. The optical properties of such a concentric structure of CSRs are not trivial to analyze.

Supplementary Note 3: Microscopic characterization of samples in Fig. 3.

The six samples of Fig. 3 in the main paper are investigated by POM in Supplementary Fig. 3, in order to assess the microscopic optical quality. We see that, although the flat films appear uniformly colored macroscopically (Fig. 3), the microscopic texture is somewhat irregular, revealing some variability in the orientation of **m** as well as several defects in the helical order development. The samples are imaged without polarizer (Supplementary Fig. 3a/g/m) and through right- (b/h/n) and left-handed (c/i/o) circular polarizers, respectively, confirming that the reflected light is indeed circularly polarized with the same handedness as the helix.

The corresponding POM images of the CSR samples are shown in the right-most three columns in Supplementary Fig. 3. As in Fig. 2h/l in the main paper, a retroreflection spot is detected at the center of each CSR in Supplementary Fig. 3d, j, p, taken without polarizers. When the sample is illuminated by right-handed circular-polarized light and analyzed through a right-handed circular polarizer in Supplementary Fig. 3e/k/q, the retroreflection remains for the right-handed CSRs with $\lambda_0^{air} \approx 0.75$ μm, while it is effectively blocked for the left-handed CSRs with green and blue retroreflection. The inverse behavior is seen for left-handed polarization (Supplementary Fig. 3f/l/r).

The green and blue retroreflection spots in Supplementary Fig. 3j/p/l/r are expected, but the central spots in Supplementary Fig. 3d/e are somewhat more surprising, since λ_0 is longer than visible light wavelengths. The weak pinkish color suggests that the spots arise from a mixture of optical phenomena. On the one hand, there is non-selective specular reflection of white light,

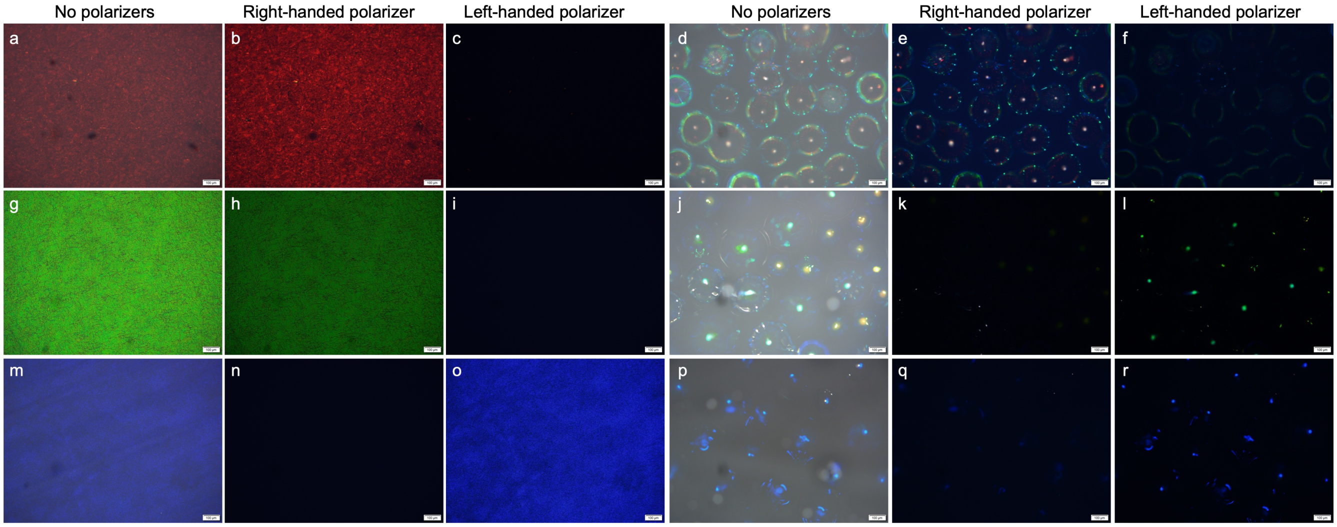

Supplementary Fig. 3. Microscopic views of flat cholesteric films and densely packed CSRs on black background. Three flat films of polymerized cholesteric liquid crystal, with red (a–c), green (g–i) and blue (m–o) retroreflection color, respectively, the helix axis **m** aligned predominantly perpendicular to the film plane, are shown in reflection microscopy, using no polarizers and circular polarizers as indicated, respectively. In the right-most three columns, three samples with close-packed CSRs in cured NOA160 glue are shown, with near-IR (d–f), green (j–l) and blue (p–r) retroreflection, respectively, for the same imaging conditions. The red and green films and the near-IR CSRs have right-handed helix while the blue film and the green and blue CSRs have left-handed helix. Scale bars: 100 μm.

which is able to resolve the helical modulation at this pitch value. The pinkish touch most likely comes from the fact that the selective reflection band extends slightly into the visible, even if λ_0 is invisible. Since the reflection band width is given by $\Delta\lambda^{LC} = p\Delta n^{nh}$, translating to $\Delta\lambda^{air} = \lambda_0^{air}\Delta n^{nh}$ in air, a typical magnitude of birefringence $\Delta n^{nh} = 0.1$ for the liquid crystals we use to make CSRs yields a reflection band width of about 75 nm for $\lambda_0^{air} \approx 0.75\ \mu$m. The short-wavelength band edge would thus be just above 700 nm, which might still be detected by the camera as a weak red color, mixing with white specular reflection to produce the pink tone.

Supplementary note 4: The PUF characteristics and rich set of challenge–response functions of CSR arrays

The unclonability and unique identity of each CSR fiducial marker is a very attractive feature, greatly increasing the trust in the use of CSR fiducial markers as a reliable means of authenticating physical objects carrying the markers and linking them to their digital twin representations. Beyond the difficulty of practically cloning a fiducial marker since this requires repeating the exact same distribution of CSRs, of different types, sizes, orientations and locations, another important feature is that the microscopic CSR marker appearance is not static, but it is very much a *function* of how the sample is illuminated and observed. This feature—which is a key component of a PUF, the 'F' standing for Function—is extremely important from a security perspective, because it makes a replica made using another material (for instance a print-out of a photo of a microscope image of the CSR fiducial) useless: at most, it might convincingly reproduce *one* appearance of the marker, but the 'dead' replica can never reproduce the way the CSR marker yields a different optical pattern (the response) if the imaging and/or observation conditions (the challenge) change. These characteristics even render simulation of the marker very difficult.

To fully demonstrate this dynamic aspect of patterns defined by CSRs we prepare a film quite densely packed with CSR shells with three different values of λ_0 as well as both helix handednesses, and we study the same area of the film using different illumination and imaging conditions, see Supplementary Fig. 4. The sample is first imaged using the same research grade POM as used in most of the images in this paper, producing panels (a–b), (e) and (i). The first two are obtained without polarizers, in reflection (a) and in transmission (b). The absence of polarizer means that all retroreflections are seen in (a), each CSR exhibiting a distinct central reflection spot, although the contrast for the lower left-hand series of deep red-reflecting CSRs is low. When circular polarizers are used, the contrast is enhanced, and then we also note the mixture of right- and left-handed CSRs in the sample. The CSRs with deep red retroreflection, almost in the IR range, are left-handed, their central spot being easy to detect in Supplementary Fig. 4i. The CSRs where the red retroreflection is easily seen in (a) turn out to be right-handed, their red spots (more orange than the previous ones) appearing with strong contrast in (e). The six blue-reflecting CSRs in (a) turn out to be divided into three right-handed, their retroreflection spots visible in (e) but not (i), and three left-handed, their spots appearing in (i) but not (e). The two green-reflecting CSRs are both right-handed, showing central spots only in (e), not in (i).

We can thus see that already the simple act of introducing a circular polarizer entirely changes the appearance of the sample, rendering a replication of the sample, such that it performs identically under any imaging condition, extremely difficult. But the same sample can exhibit another very different set of patterns, if imaged by a different type of microscope. In the remaining panels of Supplementary Fig. 4 the same area of the same sample is imaged using a digital microscope with illumination provided by a set of nine LEDs. One is mounted coaxially with the objective, corresponding to classic reflection microscopy with illumination along the viewing direction, while the remaining LEDs form a ring around the objective, illuminating the sample with a slight inclination, each in a different direction. Because the reflection of CSRs is so sensitive to the direction of illumination, each LED yields its own response in the reflection pattern.

When all nine LEDs are turned on and no polarizers are used (c), the center of each CSR exhibits not only the normal central retroreflection spot, but also an eight-fold 'flower' of spots surrounding the central spot. Of course, each 'flower' has a color determined by the pitch of the respective CSR. If circular polarizers are inserted, the 'flowers' with the wrong polarization disappear (f/j). If the central LED is turned off, only the peripheral spots of each visible flower remain (d/g/k). Finally, if only four of the surrounding LEDs are turned on, as in (h) and (l), only the corresponding four 'flower' spots are seen, the same four from each CSR that reflects the polarization let through by the polarizer.

This example illustrates how a CSR marker can appear in very different ways depending on subtle changes in the illumination and imaging conditions. Comparing (e) and (k), for instance, it can be difficult to imagine that these images were generated by the exact same sample. Each condition is a 'challenge' and the corresponding pattern is the 'response'; the number of different challenge–response combinations is practically infinite, as we can continue variations using also size and location of the illuminated area as well as the imaged area,[14,15] and we can add much more significant offsets between illumination and imaging conditions[16] than allowed by the digital microscope used in Supplementary Fig. 4. This enormous richness in the patterns that can be created by patterned CSRs makes it practically impossible to forge a certain CSR marker. On the other hand, it of course raises the demands on being able to reliably repeat the illumination and imaging conditions between the 'enrollment' phase of an authentication procedure, where the correct challenge–response combination of a certain original is

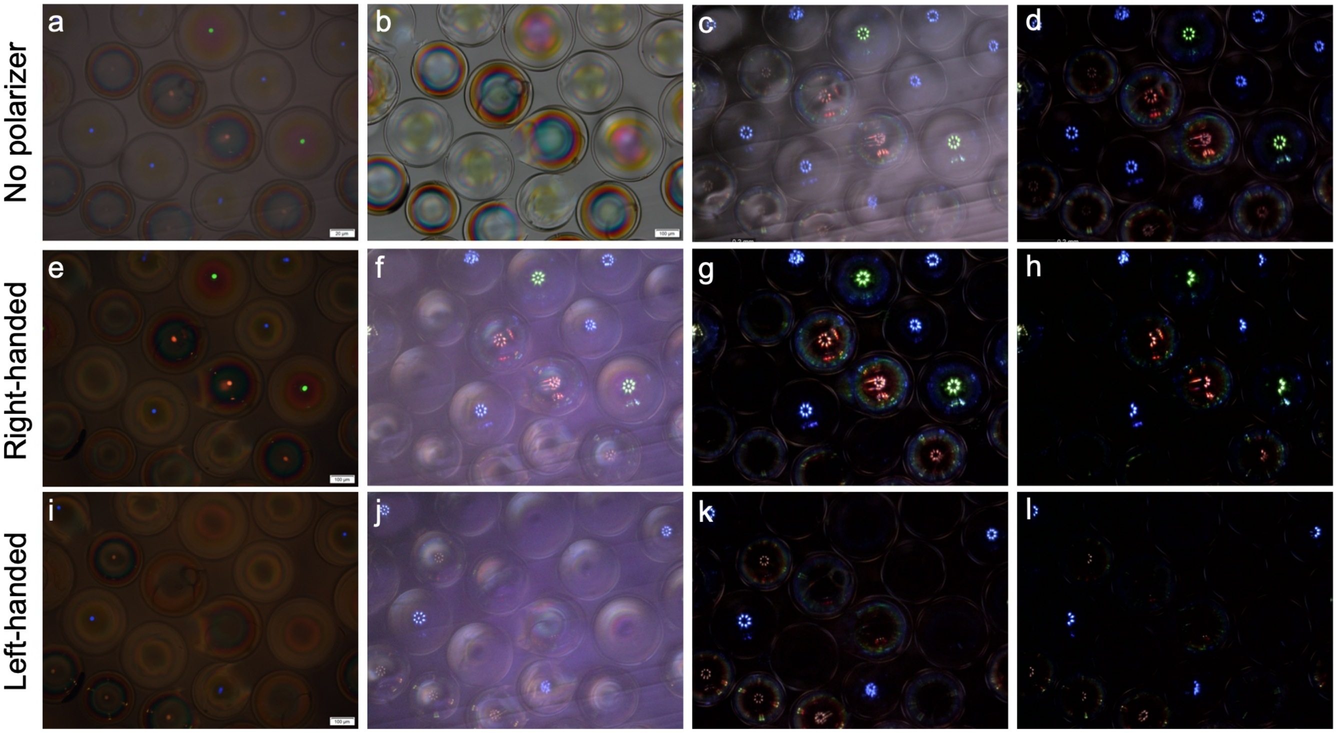

Supplementary Fig. 4. Photonic cross communication pattern between randomly arranged multicolored CSRs of both handednesses. CSR shells with red, green and blue retroreflection, respectively, some with right- and some with left-handed helix, are randomly arranged within index matching NOA160 glue and imaged using two different reflection microscopes, without polarizer (top row) and through right- (mid) and left-handed (bottom) polarizer, respectively. A research-grade Olympus BX51 polarizing optical microscope is used for photos a, e and i (reflection) and b (transmission). The remaining images are obtained in reflection with a consumer grade digital microscope (Dino-Lite AM7515MT2A) with all LEDs turned on (c, f, j), all but the central LED turned on (d, g, k) and with only four LEDs on the right side turned on (h, l). Photos c, f and j are overexposed to reveal the shell perimeters. The same area of the same sample is shown in all images, yet the appearance is very different depending on imaging and illumination conditions, illustrating the large set of challenge–response combinations. The scale bars (100 μm) in a, b, e and i apply to all panels.

recorded and stored in a reference database, and the verification phase, in which a sample of interest is tested and compared to the record stored in the database. With careful design of the hardware and of the procedure to carry out the authentication we are certain that these demands can be met, but one needs to be aware of the potential for an unexpected response in case of an uncontrolled change in illumination and/or imaging conditions.

Supplementary Note 5: Patterned deposition of CSRs that balances deterministic and random features

While traditional fiducial markers are often printed with a regular laser printer on paper, CSRs are not toner particles and the target substrate is not paper but any surface that may benefit from having a machine-readable but human-invisible fiducial marker. Moreover, the requirement to fully embed the CSRs in an index matching binder leads to further complications, in particular for CSR shells where trapped air bubbles cause strong scattering and must thus be avoided. Additionally, we wish to rapidly and reliably produce many CSR patterns that are deterministically predetermined and exactly true to their target design, in order that robots and AR devices can read them with high fidelity, yet each copy of a pattern should have its individual identity at microscopic scale, as determined by uncontrollable and unpredictable features. So how do we best produce fiducial markers based on CSRs and can the procedure be scaled up?

The fiducial markers shown in the main paper were all hand-made following a procedure that is reliable, but it is neither fast nor scalable in its present incarnation. Since we are working mainly with CSR shells, they must first be immersed in the binder (here NOA160) in a way that leaves no air bubbles. To this end, we first dispersed the shells at high density in a mixture of NOA160 and acetone, exhibiting sufficiently low viscosity that any trapped air bubbles can be removed mechanically before the

acetone is gently evaporated.[1] Next, this suspension of densely packed CSRs needs to be deposited in such a way that the CSRs cover the traditionally black areas of the target fiducial marker pattern. Our preferred way of achieving this is to first pattern the 'white' areas on the target substrate with pure NOA160, a process that can easily be scaled up using printing technologies such as pad printing (also called tampography). By UV-curing the NOA160 immediately after deposition, the 'white' areas of the marker pattern are made permanent as protrusions from the target substrate.

With the 'white' regions in place (including an additional frame around the 'black' frame that normally forms the border of a fiducial marker), the suspension of CSRs in NOA160 can be poured into the interstitial regions, any excess removed by doctor blading, and the final marker pattern is then made permanent by UV-curing. In this way, the desired fiducial marker pattern can be realized with high accuracy and reproducibility, CSRs covering 'black' regions while the 'white' regions have only NOA160 glue. By mixing CSR shells of varying size and average thickness, the variability further boosted by the intrinsic variability in size and exact geometrical features of the hole in each shell, perhaps including a few CSR beads also, the microscopic-scale identity of each marker is truly unique and in practice unclonable. If the marker detection and background subtraction is done based on retroreflection (Fig. 6 in the main paper) we can also mix equal quantities of right- and left-handed CSRs, since they appear identical to standard cameras and light sources. The random arrangement of left- and right-handed CSRs in the final marker can then be analyzed using polarization sensitive equipment for authentication, significantly boosting the unique fingerprint of each marker.

An example of the result is shown in Supplementary Fig. 5. The two markers shown next to each other in (a) have the same pattern and will appear macroscopically similar after suitable background subtraction as described above. The presence of many beads in the left marker renders it more scattering than the right one, but suitably adapted image analysis algorithms can remove this difference in the final output image. Below each marker is shown a microscopic photo as obtained by reflection POM with circular analyzer, taken from corresponding locations of each marker. Although the area macroscopically looks nearly uniform, corresponding to a 'black' fiducial marker region, the microscopic appearance is very different. In particular the random alternations of circular polarization handedness in (c) makes this texture very different from (b). Together with our computational scientist collaborators working in the field of security, we have confirmed that their existing algorithms for distinguishing unique CSR patterns from each other[17] can separate these two images very well.

Even if CSRs and binder can be optimized to each other so well that the human eye will not notice the CSRs with respect to the binder, the edge of a marker is generally easy to detect. For this reason, such edges should be avoided. In other words, the ideal binder should not only be optimized for the CSRs, but it should also be designed such that it can coat the entire target

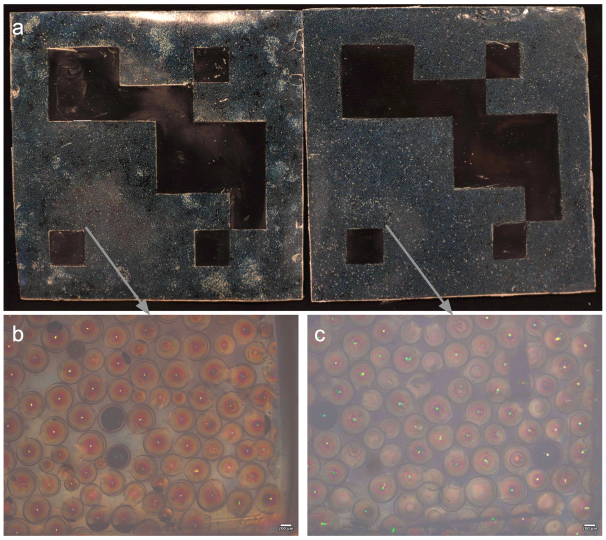

Supplementary Fig. 5. Macroscopically identical yet microscopically unique fiducial markers. a) Two fiducial markers (10 cm width) with identical pattern produced using green-reflecting CSRs, on the left mixing CSR shells and beads but all with the same handedness, on the right having almost only shells but with equal presence of right- and left-handed CSRs. **b)** Microscopic appearance of the region in the left marker in (a) at the origin of the grey arrow, as imaged by reflection POM with right-handed analyzer (the exposure is digitally enhanced in order to reveal all features with greater clarity. **c)** The corresponding POM image (digitally exposure-enhanced) of the right marker at the corresponding location, as indicated by the grey arrow, revealing that many of the CSRs in this sample have left-handed helix, thus giving no central reflection spot. Scale bars in b–c: 200 μm.

surface, even if only a small fraction of it contains CSRs. This means that the binder effectively becomes a coating for the entire product to be marked up, and then the cost of the binder becomes an important parameter to be considered. In this respect, UV-curable glues like NOA160 may not be ideal, as their large-scale application cost may be prohibitive.

To utilize CSR fiducial markers in practice, their production needs to be scaled up significantly. For very small (diameter no greater than 5 μm) CSR beads, printing technologies like pad printing are attractive solutions. For larger particles, in particular CSR shells, screen printing with large-mesh screens is a viable alternative. Serial writing with computer-programmed dispensers of CSR suspension can also be useful. By parallelizing the process with large sets of nozzles, acceptable production speeds might be reached. While many currently existing high-yield printing technologies are not immediately useful, we are confident that suitable adaptations will yield good results. Since standard inks do not have the requirements that CSR suspensions do, there has been no incentive to optimize printing technologies for CSR patterning. Once such an incentive exists, it is very much a practical engineering challenge to scale up the production, and experience shows that such challenges are overcome once sufficient motivation exists. We thus believe the most important objective at this stage is to demonstrate the usefulness of CSR fiducial markers, to maximize their signal-to-noise ratio and to minimize their visibility to the human eye.

Supplementary Note 6: A key criterion for hiding CSRs from human detection: embedding in an index-matched binder

All markers shown in this paper were made by embedding the CSRs in the UV-curable glue NOA160. This binder was chosen for two reasons. First, it is very convenient with the UV-curable nature, allowing us to place the CSRs and binder where we want them while the binder is fluid, and then make the structure permanent by exposing it to UV light. Second, the refractive index of NOA160 is $n = 1.6$, which is quite close to the average refractive index of the CSRs that we use. Strictly speaking, index matching can only be done for isotropic media, since birefringent media do not have a single refractive index to which we can match. Nevertheless, this approximate index matching is surprisingly effective in maximizing the visibility of the desired reflections at λ_0 (or other Bragg diffracted wavelengths, when photonic cross communication or shell-internal reflection is desired) and minimizing indiscriminate scattering at the many interfaces between CSRs and their surroundings.[18] This is critical, first, in order to ensure high signal-to-noise ratio, second, since the scattering can reveal the presence of the CSRs very clearly if the binder is not approaching index matching. The single hole appearing in polymerized CSR shells is very valuable from the point of view of index matching and minimization of scattering in general,[1] because it allows, first, a washing of the shell inside and outside to remove any material residues from the production process and, second, it allows the index matching binder to enter also *within* the shell, of great value for minimizing CSR visibility. Finally, having a relatively high refractive index of the binder is beneficial also in terms of minimizing undesired visible Bragg diffraction upon oblique illumination from uncontrolled light sources, because the refraction at the air–binder interface sets a maximum for the light incidence angle that can enter the binder, thereby reducing the maximum possible Bragg angle θ and thus the maximum blue-shift.[1]

To illustrate the impact of the binder refractive index, we have deposited CSRs with λ_0 in the near-UV, green and near-IR regions, respectively, on black surfaces, while they are surrounded by air, glycerol, NOA160 and NOA170, respectively. The UV-curable glue NOA170 has a slightly too large refractive index of $n = 1.7$. Each sample is imaged macroscopically with a regular DSLR camera in retroreflection configuration to represent practical usage, see Supplementary Fig. 6. When no binder is used and the CSRs are surrounded by air, those designed for near-UV and near-IR operation are both clearly visible as white spheres (Supplementary Fig. 6a/i), reflecting back the light strongly for all visible wavelengths. The green-reflecting CSRs show a predominant blueish green color in this retroreflection configuration (e), but significant white reflection is also seen. Under diffuse light, all three samples appear with easily visible white CSRs.

When the CSRs are embedded in glycerol (second column in Supplementary Fig. 6) the indiscriminate scattering goes down, saturating the green color of the CSRs in (f) somewhat and reducing the visibility of the near-UV CSRs in (j) significantly. The near-IR CSRs in (b) are still quite visible, both because of scattering and because of blue-shifted Bragg diffraction modes that now become visible as green spots. In NOA160 (third column), much of the indiscriminate scattering has disappeared, showing that the approach to index matching is reasonable even for CSRs with p long enough for light to resolve the optical modulation along the helix. Because of the reduced indiscriminate scattering, the blue-shifted Bragg diffraction from cross communication and shell-internal retroreflection now becomes significant, and almost every near-IR-CSR appears with a clear green spot. Clearly, p needs to be longer to avoid visibly colored selective reflection from near-IR CSRs in NOA160. The green and near-UV CSRs now both appear almost without any indiscriminate scattering, demonstrating the beneficial impact of optimized binder refractive index. Since the near-UV CSRs exhibit no visible Bragg diffraction, they are truly difficult to spot, whereas the green CSRs appear with a mixture of blue and green colors, arising from central retroreflection at λ_0 as well as from the blue-shifted cross communication and shell-internal retroreflection.

The slightly too large refractive index of NOA170 is interesting. It increases indiscriminate scattering due to the greater index mismatch, rendering all CSRs somewhat more visible and more white in appearance than in NOA160, even if the difference is small. However, for the near-IR CSRs it also has the effect that the green Bragg diffraction signals are almost

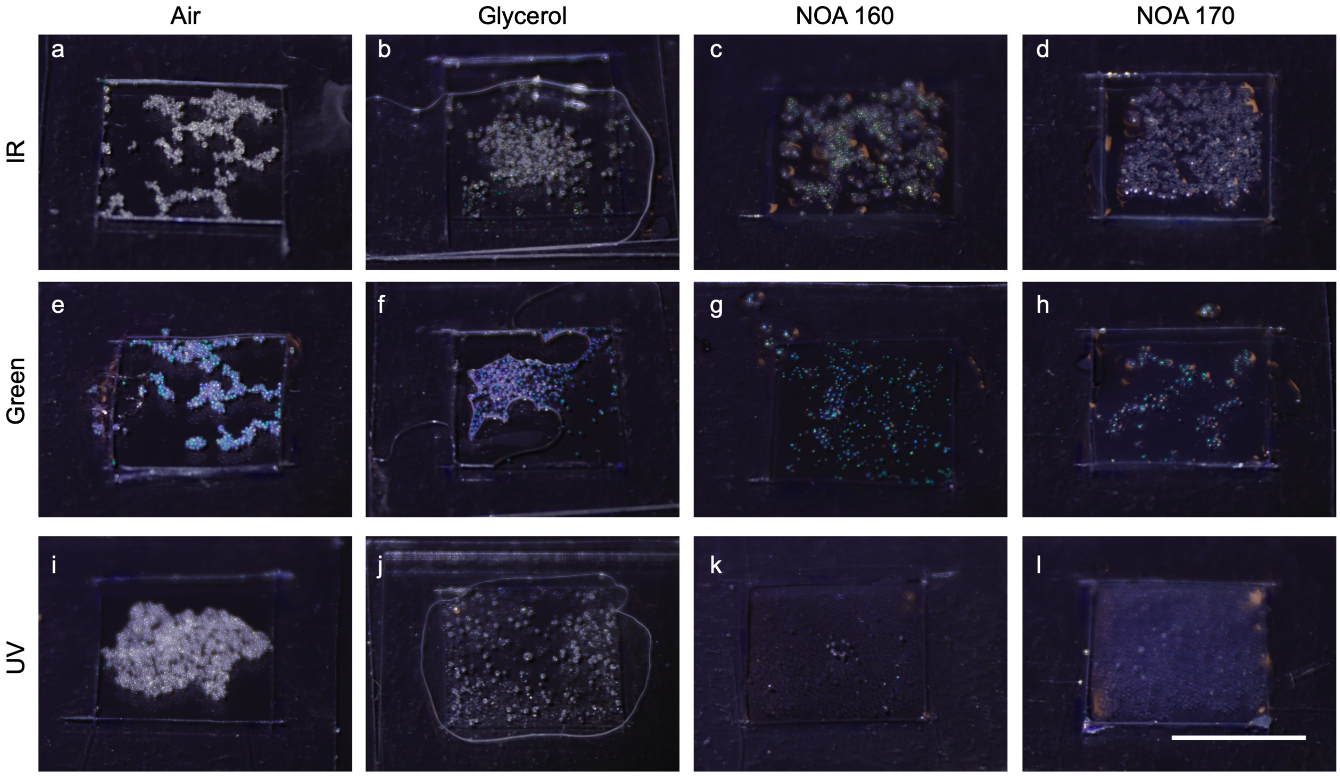

Supplementary Fig. 6. Impact of binder refractive index on CSR retroreflection. CSRs with p tuned for near-IR, green and near-UV retroreflection, respectively, surrounded by (a/e/i) air ($n = 1$), (b/f/j) glycerol ($n = 1.467$), (c/g/k) NOA160 ($n = 1.6$) and (d/h/l) NOA170 ($n = 1.7$). Scale bar: 1 cm.

absent. Also the green CSRs appear with slightly less blue color than in NOA160. We speculate that this may be due to refraction effects at the NOA170–CSR interface that are beneficial in suppressing the Bragg diffraction paths that give rise to the undesired blue-shifted reflections. Future investigations need to explore this further, but we note for now that a binder with higher refractive index than that of NOA160 may be beneficial for CSRs designed for near-IR operation.

The best way of hiding CSR fiducial markers from human detection is clearly to make the CSR–binder combination entirely invisible to the human eye, producing neither indiscriminate scattering, specular reflection or visible Bragg diffraction. While such a goal may be reached sufficiently well for certain application scenarios, in particular when utilizing CSRs designed for near-UV operation, there will surely be situations when the CSRs exhibit an unacceptable degree of visibility. As alluded to in the main paper, an alternative solution to making the CSR invisible is to *hide* them, using camouflage. In other words, even if the CSRs are visible, the human eye will not notice them if they are placed on a background that shows the same apparent color as the CSRs, as approximated in Fig. 5b in the main paper. Such a solution can be very attractive in certain contexts, but the photonic cross communication and internal shell reflection renders it more challenging to realize, in particular if the required camouflage color is red or another color in the long-wavelength end of the visible spectrum. The Technical University Eindhoven group devised a smart solution for such a conundrum: by incorporating a dye within the CSRs, undesired reflections may be suppressed by absorption in the dye.[19] Since the typical undesired Bragg diffraction modes are blue-shifted compared to λ_0, absorption of those modes does not compete with the retroreflection at λ_0, which is normally the desired mode.

Supplemental Bibliography

1. Yong Geng, Rijeesh Kizhakidathazhath, and Jan P. F. Lagerwall. Encoding hidden information onto surfaces using polymerized cholesteric spherical reflectors. *Adv. Funct. Mater.*, 31(21):2100399, 2021.

2. Anjali Sharma and Jan P. F. Lagerwall. Influence of head group and chain length of surfactants used for stabilising liquid crystal shells. *Liq. Cryst.*, 45(13-15):2319–2328, 2018.

3. A. Sharma, A. M. Stoffel, and J. P. F. Lagerwall. Liquid crystal elastomer shells with topological defect-defined actuation: Complex shape morphing, opening/closing, and unidirectional rotation. *J. Appl. Phys.*, 129(17):174701, 2021.

4. A Utada, E. Lorenceau, D. R. Link, P. D. Kaplan, H. A. Stone, and D. A. Weitz. Monodisperse double emulsions generated from a microcapillary device. *Science*, 308(5721):537–541, 2005.

5. Yong Geng, JungHyun Noh, Irena Drevensek-Olenik, Romano Rupp, Gabriele Lenzini, and Jan P. F. Lagerwall. High-fidelity spherical cholesteric liquid crystal bragg reflectors generating unclonable patterns for secure authentication. *Sci. Rep.*, 6:26840, 2016.

6. Kunyun He, Francisco Campo-Cortés, Martyna Goral, Teresa López-León, and José Manuel Gordillo. Micron-sized double emulsions and nematic shells generated via tip streaming. *Physical Review Fluids*, 4(12):124201, 2019.

7. Christina Schütz, Johanna R. Bruckner, Camila Honorato-Rios, Zornitza Tosheva, Manos Anyfantakis, and Jan P. F. Lagerwall. From equilibrium liquid crystal formation and kinetic arrest to photonic bandgap films using suspensions of cellulose nanocrystals. *Crystals*, 10(3):199, 2020.

8. Bruno Frka-Petesic, Gen Kamita, Giulia Guidetti, and Silvia Vignolini. Angular optical response of cellulose nanocrystal films explained by the distortion of the arrested suspension upon drying. *Physical Review Materials*, 3(4):045601, 2019.

9. Sung Tae Kim and Heino Finkelmann. Cholesteric liquid single-crystal elastomers (lsce) obtained by the anisotropic deswelling method. *Macromol. Rapid Commun.*, 22(6):429–433, 2001.

10. M Humar and I Musevic. 3d microlasers from self-assembled cholesteric liquid-crystal microdroplets. *Opt. Express*, 18(26):26995–27003, 2010.

11. T Yang, D Yuan, W Liu, Z Zhang, K Wang, Y You, H Ye, LT de Haan, Z Zhang, and G Zhou. Thermochromic cholesteric liquid crystal microcapsules with cellulose nanocrystals and a melamine resin hybrid shell. *ACS Appl Mater Interfaces*, 14(3):4588–4597, 2022.

12. SS Lee, HJ Seo, YH Kim, and SH Kim. Structural color palettes of core-shell photonic ink capsules containing cholesteric liquid crystals. *Adv. Mater.*, 29(23):1606894, 2017.

13. S Park, SS Lee, and SH Kim. Photonic multishells composed of cholesteric liquid crystals designed by controlled phase separation in emulsion drops. *Adv. Mater.*, 32(30):e2002166, 2020.

14. Yong Geng, JungHyun Noh, Irena Drevensek-olenik, Romano Rupp, and Jan P F Lagerwall. Elucidating the fine details of cholesteric liquid crystal shell reflection patterns. *Liq. Cryst.*, 44(12-13):1948–1959, 2017.

15. JungHyun Noh, Hsin-Ling Liang, Irena Drevensek-Olenik, and Jan P. F. Lagerwall. Tuneable multicoloured patterns from photonic cross communication between cholesteric liquid crystal droplets. *J. Mater. Chem. C*, 2(5):806–810, 2014.

16. M Schwartz, G Lenzini, Y Geng, PB Rønne, PYA Ryan, and JPF Lagerwall. Cholesteric liquid crystal shells as enabling material for information-rich design and architecture. *Adv. Mater.*, 30(30):1707382, 2018.

17. Mónica P. Arenas, Hüseyin Demirci, and Gabriele Lenzini. An analysis of cholesteric spherical reflector identifiers for object authenticity verification. *Machine Learning and Knowledge Extraction*, 4(1):222–239, 2022.

18. E. Beltran-Gracia and O. L. Parri. A new twist on cholesteric films by using reactive mesogen particles. *J. Mater. Chem. C*, 3(43):11335–11340, 2015.

19. A Belmonte, T Bus, DJ Broer, and APHJ Schenning. Patterned full-color reflective coatings based on photonic cholesteric liquid-crystalline particles. *ACS Appl Mater Interfaces*, 11(15):14376–14382, 2019.
